# Sequence Determinants of TDP-43 Ribonucleoprotein Condensate Formation and Axonal Transport in Neurons

**DOI:** 10.3389/fcell.2022.876893

**Published:** 2022-05-12

**Authors:** Sonali S. Vishal, Denethi Wijegunawardana, Muthu Raj Salaikumaran, Pallavi P. Gopal

**Affiliations:** ^1^ Department of Pathology, Yale School of Medicine, New Haven, CT, United States; ^2^ Program in Cellular Neuroscience, Neurodegeneration, and Repair, Yale School of Medicine, New Haven, CT, United States

**Keywords:** TDP-43, ribonucleoprotein granules, biomolecular condensates, neuron, axonal transport, amyotrophic lateral sclerosis

## Abstract

Mutations in TDP-43, a RNA-binding protein with multiple functions in RNA metabolism, cause amyotrophic lateral sclerosis (ALS), but it is uncertain how defects in RNA biology trigger motor neuron degeneration. TDP-43 is a major constituent of ribonucleoprotein (RNP) granules, phase separated biomolecular condensates that regulate RNA splicing, mRNA transport, and translation. ALS-associated TDP-43 mutations, most of which are found in the low complexity domain, promote aberrant liquid to solid phase transitions and impair the dynamic liquid-like properties and motility of RNP transport granules in neurons. Here, we perform a comparative analysis of ALS-linked mutations and TDP-43 variants in order to identify critical structural elements, aromatic and charged residues that are key determinants of TDP-43 RNP transport and condensate formation in neurons. We find that A315T and Q343R disease-linked mutations and substitutions of aromatic residues within the α-helical domain and LARKS, show the most severe defects in TDP-43 RNP granule transport and impair both anterograde and retrograde motility. F313L and F313-6L/Y substitutions of one or both phenylalanine residues in LARKS suggest the aromatic rings are important for TDP-43 RNP transport. Similarly, W334F/L substitutions of the tryptophan residue in the α-helical domain, impair TDP-43 RNP motility (W334L) or anterograde transport (W334F). We also show that R293A and R293K mutations, which disrupt the only RGG in the LCD, profoundly reduce long-range, directed transport and net velocity of TDP-43 RNP granules. In the disordered regions flanking the α-helical domain, we find that F283Y, F397Y or Y374F substitutions of conserved GF/G and SYS motifs, also impair anterograde and/or retrograde motility, possibly by altering hydrophobicity. Similarly, ALS-linked mutations in disordered regions distant from the α-helical domain also show anterograde transport deficits, consistent with previous findings, but these mutations are less severe than A315T and Q343R. Overall our findings demonstrate that the conserved α-helical domain, phenylalanine residues within LARKS and RGG motif are key determinants of TDP-43 RNP transport, suggesting they may mediate efficient recruitment of motors and adaptor proteins. These results offer a possible mechanism underlying ALS-linked TDP-43 defects in axonal transport and homeostasis.

## Introduction

Amyotrophic lateral sclerosis (ALS) is a fatal disease of motor neuron degeneration (Hardiman et al., 2017). Nearly all (97%) ALS cases show pathologic aggregation and nuclear depletion of TDP-43 (transactive response DNA-binding protein of 43 kDa), a highly conserved, ubiquitously expressed DNA/RNA-binding protein which regulates post-transcriptional RNA processing and mRNA stability, translation, and transport (Neumann et al., 2006; Ling et al., 2013; Taylor et al., 2016). TDP-43 is comprised of a multimer-forming N-terminal domain (NTD; 1–106) which contains a nuclear localization signal (Mompean et al., 2016; Afroz et al., 2017); two RNA recognition motif domains (RRM1 & RRM2; 107–262) (Lukavsky et al., 2013); and a predominantly disordered C-terminal low complexity domain (LCD, 263–414) enriched in glycine, glutamine, and asparagine residues (Buratti et al., 2005; Fuentealba et al., 2010; Lim et al., 2016). The discovery of mutations in the gene encoding TDP-43 (*TARDBP*), in familial and rare sporadic cases of ALS provided a critical link between TDP-43 genetic variants, pathology, and ALS pathogenesis (Gitcho et al., 2008; Kabashi et al., 2008; Sreedharan et al., 2008; Corrado et al., 2009). Thus, human genetic data and pathologic evidence highlight altered RNA metabolism as a critical pathogenic mechanism of neurodegeneration involving TDP-43 loss of nuclear function, toxic gain of function, or both.

Under physiologic conditions, TDP-43 plays an essential role in pre-mRNA splicing, repression of cryptic exons, and mRNA stability (Polymenidou et al., 2011; Ling et al., 2015). TDP-43 binds to consensus UG_n_ repeats in introns and the 3′UTR of thousands of transcripts, including transcripts important for synaptic and axonal homeostasis (Polymenidou et al., 2011; Tollervey et al., 2011). TDP-43 and other disease linked RNA-binding proteins are constituents of ribonucleoprotein (RNP) condensates, dynamic non-membrane bound cellular compartments that assemble through liquid-liquid phase separation (LLPS) (Banani et al., 2017; Shin and Brangwynne, 2017). Examples of RNP condensates include nucleoli and Cajal bodies as well as stress granules and neuronal RNA transport granules in the cytoplasm (Brangwynne et al., 2011; Courchaine et al., 2016; Banani et al., 2017). RNP condensates containing TDP-43 regulate pre-mRNA splicing and mRNA translation, both important for spatiotemporal regulation of transcripts and neuronal function (Altman et al., 2021; Gao et al., 2021; Hallegger et al., 2021). TDP-43 is a component of neuronal RNP granules that transport critical mRNAs in the axon and dendrites (Fallini et al., 2012; Alami et al., 2014; Liu-Yesucevitz et al., 2014). Furthermore, we have shown that TDP-43 displays dynamic, liquid-like biophysical properties of condensates in primary cortical neurons and that ALS-linked mutations fundamentally perturb the biophysical properties of these condensates (Gopal et al., 2017).

Purified full-length TDP-43 undergoes LLPS, and the TDP-43 LCD domain is also able to phase separate at physiologic salt concentrations and in the presence of RNA *in vitro* (Conicella et al., 2016). Multiple elegant studies have demonstrated that electrostatic and cation-pi interactions drive phase separation of several RNA-binding proteins, including FUS, DDX4, and hnRNPA1 *in vitro* and in cells (Kato et al., 2012; Molliex et al., 2015; Nott et al., 2015). For example, phase separation of purified FUS depends on multiple tyrosine residues in the prion like domain (PLD) which interact with arginine residues in the RNA binding domain (RBD) (Wang et al., 2018). In contrast, TDP-43 phase separation depends on a short stretch of conserved amino acids in LCD (320–340) that forms an α-helical domain and serves as a backbone to drive LLPS (Conicella et al., 2016). The α-helical domain is flanked by two intrinsically disordered regions (IDR1: 274–316 and IDR2: 341–414) which contain LLPS (GFG/FG) motifs, tryptophan residues and a single tyrosine residue that also influence phase separation *in vitro* (Conicella et al., 2016; Li et al., 2018b; Schmidt et al., 2019; Pantoja-Uceda et al., 2021). In addition, IDR1 contains a low complexity, aromatic-rich, kinked segment (LARKS) at 312–317, which has been shown to mediate reversible adhesion between the LCDs of RNA binding proteins (Guenther et al., 2018; Hughes et al., 2018). The contribution of aromatic residues, LARKS, GFG/FG, and RGG motifs to phase separation *in vivo*, specifically partitioning of TDP-43 into neuronal RNP condensates and RNP transport, are not well understood.

Several studies suggest that aberrant phase transitions of TDP-43 and other disease linked RNA-binding proteins are important in ALS pathophysiology (Molliex et al., 2015; Patel et al., 2015; Conicella et al., 2016; McGurk et al., 2018). Most ALS-linked TDP-43 mutations cluster within the LCD but display different effects on LLPS. A few disease-associated mutants, including A321V and G335D enhance LLPS, whereas A321G, Q331K, and M337V reduce LLPS by disrupting the structure of the α-helical region or by reducing helix-helix inter-molecular interactions (Conicella et al., 2016; Li et al., 2018a; Li et al., 2018b; Conicella et al., 2020). In addition, TDP-43 mutations within LARKS may enhance formation of irreversible protein aggregates. Apart from LARKS, TDP-43 LCD contains six short stretches of amino acids called steric zippers that also may play a role in TDP-43 aggregation (Guenther et al., 2018). Relatively few ALS-linked mutations have been assessed for defects in splicing regulation, mRNA localization, and translation *in vivo* (D'Ambrogio et al., 2009; Alami et al., 2014; Gopal et al., 2017; Roczniak-Ferguson and Ferguson, 2019; Conicella et al., 2020).

Although recent work suggests assembly into biomolecular condensates may modulate the RNA-regulatory functions of TDP-43 and other constituents of RNP condensates, conflicting data exist, and many questions about the physiologic function of condensates in neurons and their pathophysiology in disease remain (Schmidt et al., 2019; Conicella et al., 2020; Altman et al., 2021; Gao et al., 2021; Hallegger et al., 2021). Inspired by elegant work that uncovered molecular determinants of TDP-43 LLPS and fibrillization *in vitro* (Conicella et al., 2016; Li et al., 2018b; Schmidt et al., 2019; Pantoja-Uceda et al., 2021), in the present study we sought to determine whether 1) different ALS-linked TDP-43 mutations or 2) disruption of specific aromatic and charged residues in the LCD have distinct effects on the formation of TDP-43 positive RNP condensates and their functional impact on TDP-43 RNP transport in neurons.

## Materials and Methods

### Plasmids and Antibodies

Constructs include eGFP-TDP-43 WT (gift from Dr. Virginia Lee, University Pennsylvania, Philadelphia), DsRed2-mito (gift from Dr. Thomas Schwarz). To generate different ALS associated TDP-43 mutations and mutations at aromatic and charged residues, site directed mutagenesis was used. Primers used to generate these mutants are given in Supplementary Table S1. For western blot, primary antibodies against TDP-43 (Biolegend, TDP2H4, 1:1000) and betaIII Tubulin (Promega, G7121, 1:1000) were used. IRDye labelled secondary antibodies from LI-COR were used.

### Cell Culture and Transfection

Primary cortical neurons were dissociated from E18 Sprague Dawley rat embryos and suspended in Neurobasal medium (Gibco), as described previously (Gopal et al., 2017). Neurons were plated at a density of 150,000 cells/ml on poly L-lysine (0.5 mg/ml; Sigma) coated glass bottom dishes (MatTek) and were maintained in Neurobasal medium (Gibco) containing B27 supplement (Invitrogen), 2 mM GlutaMAX (Gibco), 33 mM glucose (Gibco) and 100 units/ml penicillin with 100 μg/ml streptomycin at 37°C with 5% CO_2_. On the third day *in vitro* (DIV), 1 µM AraC was added. Neurons (DIV6-9) were co-transfected with DsRed-Mito and eGFP tagged WT TDP-43 or mutant TDP-43 in the ratio of 1:1.5, using lipofectamine 2000 (Invitrogen). HeLa TDP-43 knockout (KO) and KO with TDP-43 rescue cell lines (gift from Dr. Shawn Ferguson, Yale University, New Haven) were cultured in DMEM containing 10% FBS and 1% Glutamax and were transfected using Fugene (4:1 ratio with DNA).

### Live Cell Imaging

Live imaging of primary cortical neurons (DIV7-10) was performed 16 h post-transfection in Hibernate E (Brainbits) supplemented with 2% B27 and 2 mM GlutaMAX, in a temperature-controlled chamber mounted on an inverted NikonTi microscope with apochromat 63x1.49 NA oil-immersion objectives; images were acquired on a Perkin Elmer UltraVIEW VOX spinning disk confocal system equipped with an Ultraview Photokinesis (Perkin Elmer) unit and a C9100-50 EMCCD camera (Hamamatsu) controlled by Volocity software (Perkin Elmer). Axons were identified by morphologic criteria and were imaged at one frame every 2s for 5 min. Fluorescence recovery after photobleaching (FRAP) images were acquired using the 488 nm laser at 1 frame/second for 3–5 s prior to and for 120 s subsequent to photobleaching. The Photokinesis unit was calibrated prior to each experiment, as described (Gopal et al., 2017).

### Image and Data Analysis

All image processing and analysis was performed using ImageJ/Fiji and/or custom, automated analyses in MATLAB R2018a (Natick, Massachusetts). For TDP-43 motility, kymographs were prepared in Fiji, and analyzed using custom MATLAB programs (Gopal et al., 2017; Guedes-Dias et al., 2019) to calculate net velocities, net and cumulative displacement, run lengths, and fraction of motile, oscillatory, and stationary TDP-43 RNP granules. TDP-43 granules that underwent long range directional transport, defined as net displacement of ≥10 μm in 5 min or less, were defined as “motile”. Motile granules were classified further by net direction of transport (anterograde or retrograde). TDP-43 granules that were transported <10 μm were considered “non-motile”; this group of granules was further broken down into two sub-groups: stationary and oscillatory granules. Stationary TDP-43 granules were defined by < 5 μm cumulative displacement and oscillatory granules displayed > 5 μm cumulative displacement.

**FIGURE 1:**
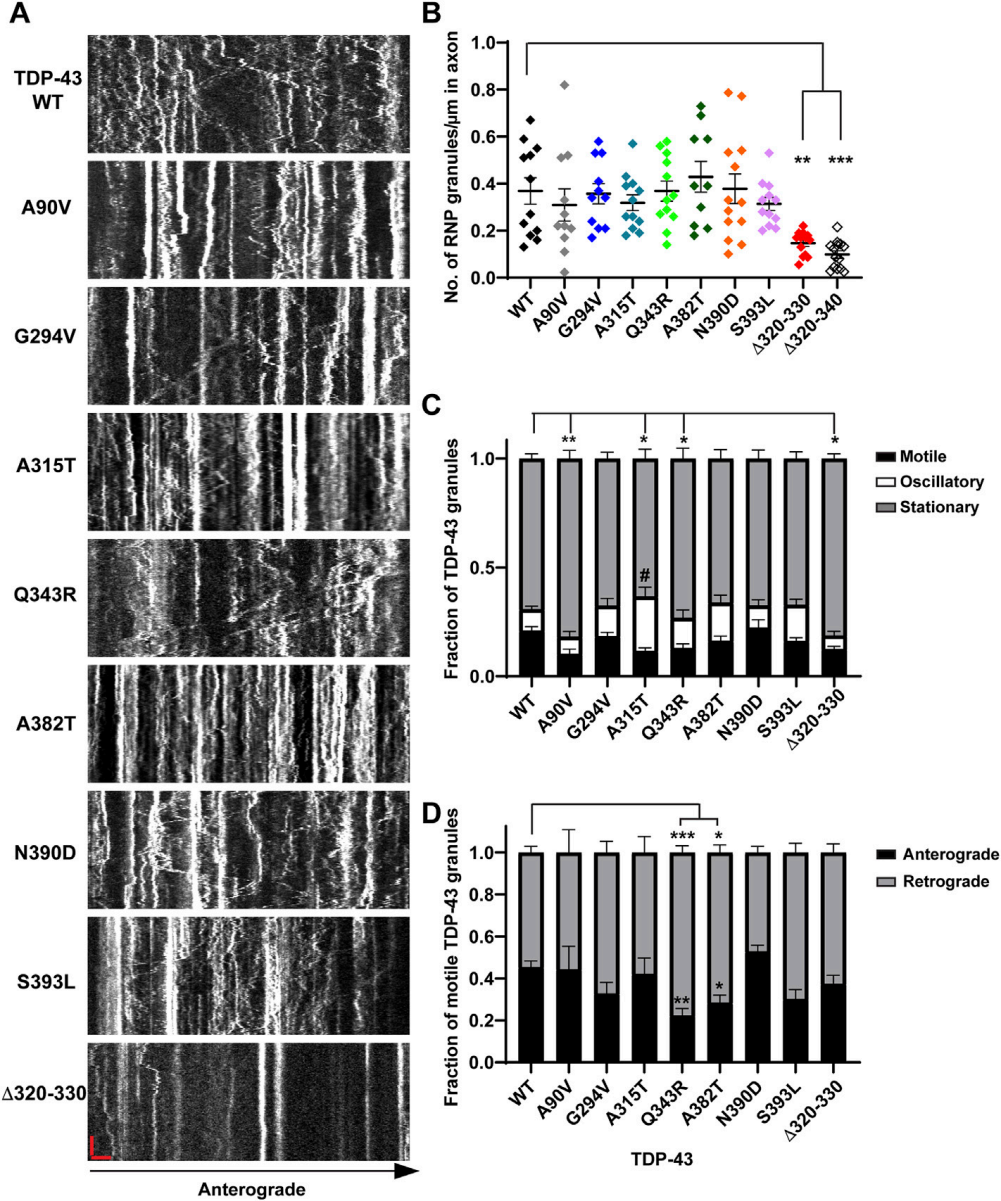
Neuronal RNP granules composed of ALS-linked TDP-43 mutants or TDP-43 Δ320-330 exhibit reduced motility. **(A)** Axons of primary cortical neurons (DIV7-10) expressing GFP tagged WT or mutant TDP-43 were imaged using live-cell spinning disk confocal microscopy. Representative kymographs of GFP tagged TDP-43 WT or mutant RNP granules are shown. In the kymographs, the retrograde direction (towards the Soma) is to the left and the anterograde direction is to the right. The scale bar (horizontal red line) corresponds to 3 μm (*x* axis) and time scale (vertical red line) corresponds to 50 s (*y* axis). **(B)** The number of TDP-43 RNP granules per μm length of axon for TDP-43 WT and different mutants is shown. TDP-43 Δ320-330 and Δ320-340 lack part or all of the α-helical domain, respectively, and serve as controls expected to substantially reduce phase separation. For each condition, *n* = 10–14 neurons were analyzed from 3 independent experiments (*N* = 3). **(C)** Fraction of TDP-43 WT or mutant RNP granules that are stationary, oscillatory, or motile in the axon; motile puncta ≥10 μm net displacement in 5 min. **(D)** The fraction of motile TDP-43 RNP granules for WT and mutants (shown as black bars in Figure 1C), is further categorized by retrograde or anterograde motility. For each condition, *n* = 12–14 neurons were analyzed from 3 independent experiments (*N* = 3). Error bars represent standard error mean. One way ANOVA (Kruskal–Wallis test with Dunn’s correction for multiple comparisons) was used to determine statistically significant differences among samples; #*p* < 0.1, *p < 0.05, **p < 0.01, and ***p < 0.0005.

Quantification of the number of TDP-43 RNP granules in the axon was performed in a semi-automated manner using ImageJ/Fiji software. Briefly, a single time point (t75) image was extracted from each live imaging movie, the axon was straightened and the image was converted to 8 bit. Detection of TDP-43 WT and mutant granules was performed by auto-thresholding with default settings, followed by application of built-in method for local maxima detection (prominence setting of 25). The number of TDP-43 RNP granules thus obtained was divided by the length of the axon in microns; these data are shown as mean ± SEM (Figure 1), without matching of expression level. Quantification of TDP-43 RNP granule formation while also accounting for expression level was performed as follows. After subtraction of background and maxima intensity, the mean cytoplasmic fluorescence intensity in the straightened axon was calculated as the average of mean gray values across the entire length of the axon. The number of TDP-43 RNP granules per micron was then plotted as a function of mean axonal cytoplasmic fluorescence intensity, which served as a rough estimate of expression level. For FRAP analysis, mean fluorescence intensities from three regions of interest (ROIs) were obtained: 1) photobleached granule, 2) an unbleached region (used to correct for overall photobleaching), and 3) background. A background subtraction was performed, the intensities of the photobleached ROI were normalized from 0 to 1 (pre-photobleach intensity = 1) and corrected for overall photobleaching. Corrected normalized fluorescence intensities were plotted as a function of time.

### Conservation Score Analysis

The ConSurf tool (Ashkenazy et al., 2010) was used to compute conservation scores. TDP-43 sequences from 47 eukaryotic species were retrieved from the Uniprot database (Supplementary Table S2). The conservation scale was divided into nine levels, one to nine. A conservation grade of 1 implies that the sequences are varied, whereas a grade of 9 indicates that the residues are the most conserved. Furthermore, we predicted the buried, exposed, structurally, and functionally important residues in TDP-43 using the Conseq tool (Berezin et al., 2004).

### Statistics

Statistical tests were performed in GraphPad Prism. A Student’s t-test (for normally distributed data) or Mann-Whitney U test (for non-normally distributed data) were used to compare two groups, and one-way analysis of variation with Tukey’s post-hoc test was used to compare multiple groups of normally distributed data. The Kruskal–Wallis test with Dunn’s correction was performed to compare multiple groups of non-normally distributed data. Sample sizes were chosen based on reported data from similar studies in the literature for the neuronal live imaging field. Figure legends state *n*, the number of neurons or granules per condition; N represents the number of times the experiment was independently repeated.

## Results

### TDP-43 A315T, Q343R, and LLPS Mutants Exhibit a Reduced Fraction of Motile RNP Granules

Prior work has shown that the TDP-43 LCD is necessary for heterotypic interactions and assembly into stress granules (Buratti et al., 2005; D'Ambrogio et al., 2009; Bentmann et al., 2012). The conserved α-helical region mediates helix-helix contacts, with additional contributions from glycine-rich regions and aromatic residues, to drive TDP-43 LLPS (Conicella et al., 2016; Li et al., 2018b). Therefore, we examined condensate formation and transport of TDP-43 α-helical domain mutants (Δ320-330, Δ320-340), which served as controls expected to substantially reduce LLPS. In addition, we asked whether ALS-linked mutations adjacent to or within the conserved alpha-helical region (A315T, Q343R) show more severe axonal transport defects than mutations distant from the conserved region (G294V, A382T, N390D, and S393L); see Supplementary Figure S1. The NLS associated A90V variant served as a negative control that has an intact LCD, but mis-localizes to the cytoplasm, similar to several ALS-linked mutants, including G294V, A315T and A382T (Buratti, 2015). A summary of these mutants is given in Supplementary Table S3. To address these questions, we performed live cell confocal imaging of rodent primary cortical neurons expressing eGFP-tagged human TDP-43 WT (or mutants) and DsRed-Mito. Semi-automated quantitative image analysis was used to study TDP-43 motility, dynamics, and the number of TDP-43 positive RNP condensates in the axon. Using this approach in prior work, we have shown that exogenous expression of TDP-43 mirrors endogenous TDP-43, with predominantly nuclear localization and punctate dendritic and axonal expression (Gopal et al., 2017). Furthermore, we demonstrated that TDP-43 positive RNP granules in the axon display liquid-like biophysical properties characteristic of biomolecular condensates, suggesting that TDP-43 partitions into neuronal RNP condensates through phase separation.

Consistent with structural data and recent functional studies examining the role of the α-helical domain (Conicella et al., 2020; Hallegger et al., 2021), indeed we found that TDP-43 mutant lacking part of the α-helical domain (Δ320–330) forms a highly reduced number of TDP-43 positive RNP granules in the axon (0.15 ± 0.01 granules/μm) compared to WT (0.37 ± 0.01 granules/μm), despite similar expression level (Figures 1A,B; Supplementary Figures S2A–D). In further support of this observation, simple linear regression analysis demonstrates a positive correlation between the number of TDP-43 WT RNP granules per axon length as a function of expression level (*R* ^2^ = 0.76; slope = 0.017 ± 0.002); in contrast, the slope of this correlation is significantly reduced for TDP-43 Δ320-330 (slope = 0.003 ± 0.001; *p* < 0.001; Supplementary Figure S2C). In addition to reduced formation of condensates overall, TDP-43 Δ320–330 mutant also shows a lower fraction of motile RNP granules, defined as granules transported net distance ≥10 μm (Δ320–330 motile fraction: 0.13 ± 0.01) compared to that of WT TDP-43 (0.21 ± 0.02, *p* < 0.05; Figures 1A,C). As expected, TDP-43 Δ320–340 shows a more profound reduction in RNP condensate formation in the axon, to the extent that many neurons have very few detectable granules (0.10 ± 0.02 granules/μm, *p* < 0.001; Figure 1B). Consequently, the motility of TDP-43 Δ320-340 RNP condensates could not be analyzed in a robust manner, but limited data suggest that TDP-43 Δ320-340 also shows a reduced motile fraction (not shown).

**FIGURE 2:**
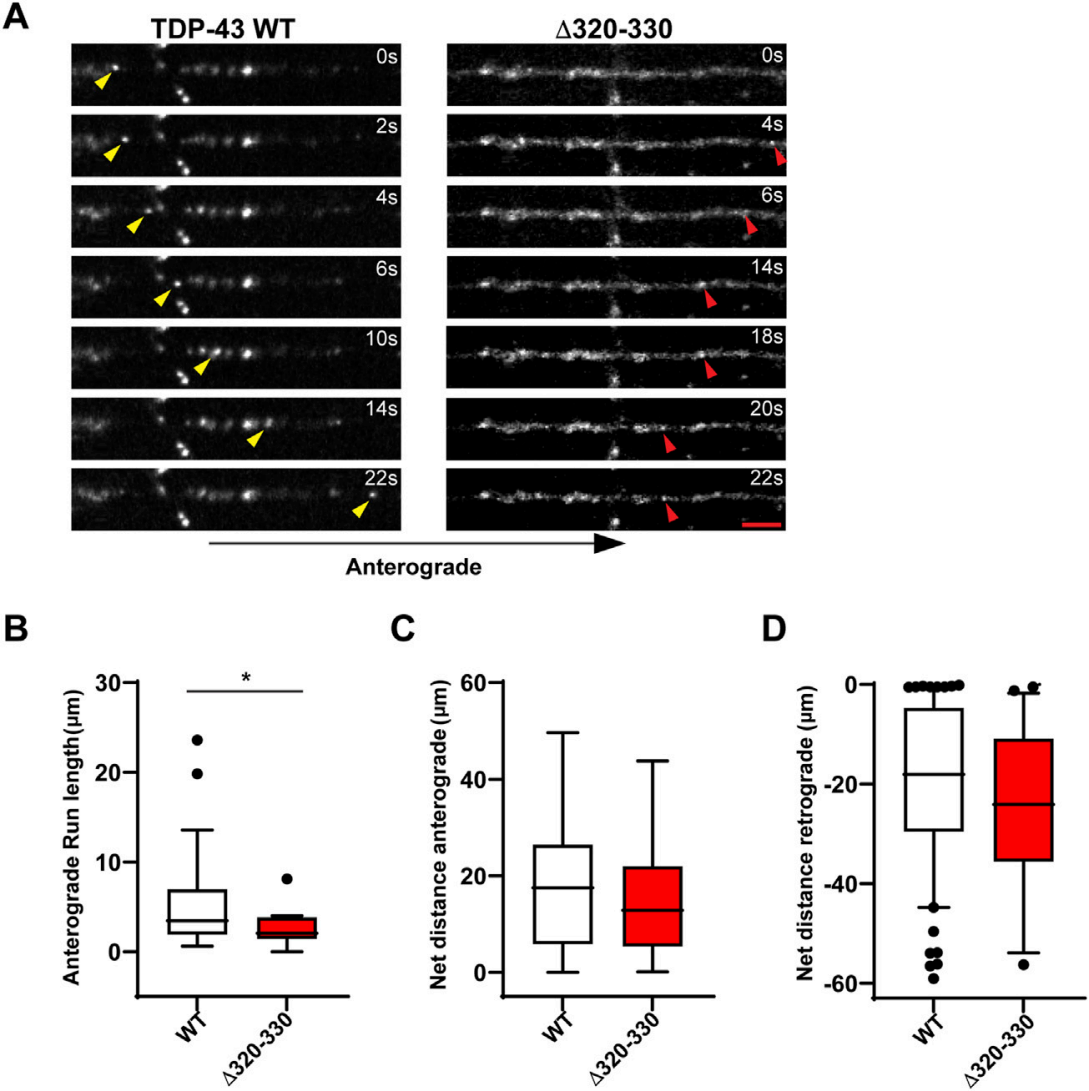
Disrupting the α-helical domain impairs anterograde motility. **(A)** Time-lapse images of a representative motile TDP-43 WT RNP granule (left, yellow arrowhead) travelling in the anterograde direction and TDP-43 Δ320-330 RNP granule moving retrograde (right, red arrowhead); scale bar: 3 μm. **(B)** TDP-43 WT RNP granules exhibit significantly longer anterograde run lengths (μm) than RNP granules containing TDP-43 Δ320-330. **(C)** The net distance (μm) anterograde or **(D)** retrograde travelled by motile WT and Δ320-330 TDP-43 RNP granules. The values are from the 3 independent experiments (WT *n* = 134 and Δ320-330 *n* = 36); plotted data represent mean ± SEM. Mann-Whitney test was used to determine statistical significance, *p < 0.05.

Closer analysis of motile RNP granules containing TDP-43 Δ320-330 revealed no change in the fraction of anterograde and retrograde tracks (Figure 1D). However, TDP-43 Δ320-330 granules display decreased anterograde run lengths and a trend towards lower net anterograde transport in the axon (Figures 2A–C). Net distance in the retrograde direction was not affected (Figure 2D). Combined with prior studies showing that α-helical domain mutants disrupt LLPS *in vitro*, our data suggest TDP-43 α-helical domain mutants are less efficiently recruited to neuronal RNP condensates containing functional components required for motility, particularly in the anterograde direction.

In contrast to α-helical domain mutations, the ALS-linked mutations we studied do not significantly alter the number of TDP-43 positive RNP granules in the axon (Figures 1A,B). For most ALS-linked mutations, simple linear regression analysis of TDP-43 RNP granule number per axon length as a function of expression level shows a similar positive correlation and slope as TDP-43 WT (Supplementary Figures S2E,F). However, the slope of this relationship is slightly less for TDP-43 Q343R (*p* < 0.05) and trends lower for A315T (*p* = 0.06), compared to the slope observed for WT TDP-43 (Supplementary Figure S2F). The significance of this finding is not clear, however, and could represent subtle defects in the formation of TDP-43 positive condensates and/or the presence of fewer, larger aggregate-like structures with these mutants.

Intriguingly, mutations of A315 and Q343, residues which participate in minor populations of short helical conformations adjacent to the conserved α-helical domain (Conicella et al., 2016), show similar effects as Δ320-330 on TDP-43 RNP motility in the axon. We found that granules containing TDP-43 A315T show a significantly lower motile fraction (0.12 ± 0.01; *p* < 0.01) compared to WT (Figures 1A,C). This reduction in the motile fraction is accompanied by a trend towards increased oscillatory motility (Figure 1C, #*p* = 0.05), suggesting RNP granules containing A315T are not completely immotile but instead show defects in long-range, directed motility. A lower motile fraction is also observed with RNP granules containing TDP-43 Q343R mutant (0.13 ± 0.02, *p* < 0.05; Figure 1C). Unexpectedly, we also found that RNP granules containing the NLS variant A90V show a significantly lower motile fraction compared to those containing WT TDP-43 (Figure 1C).

### ALS-Linked TDP-43 LCD Mutants Show Defects in Anterograde Transport

**FIGURE 3:**
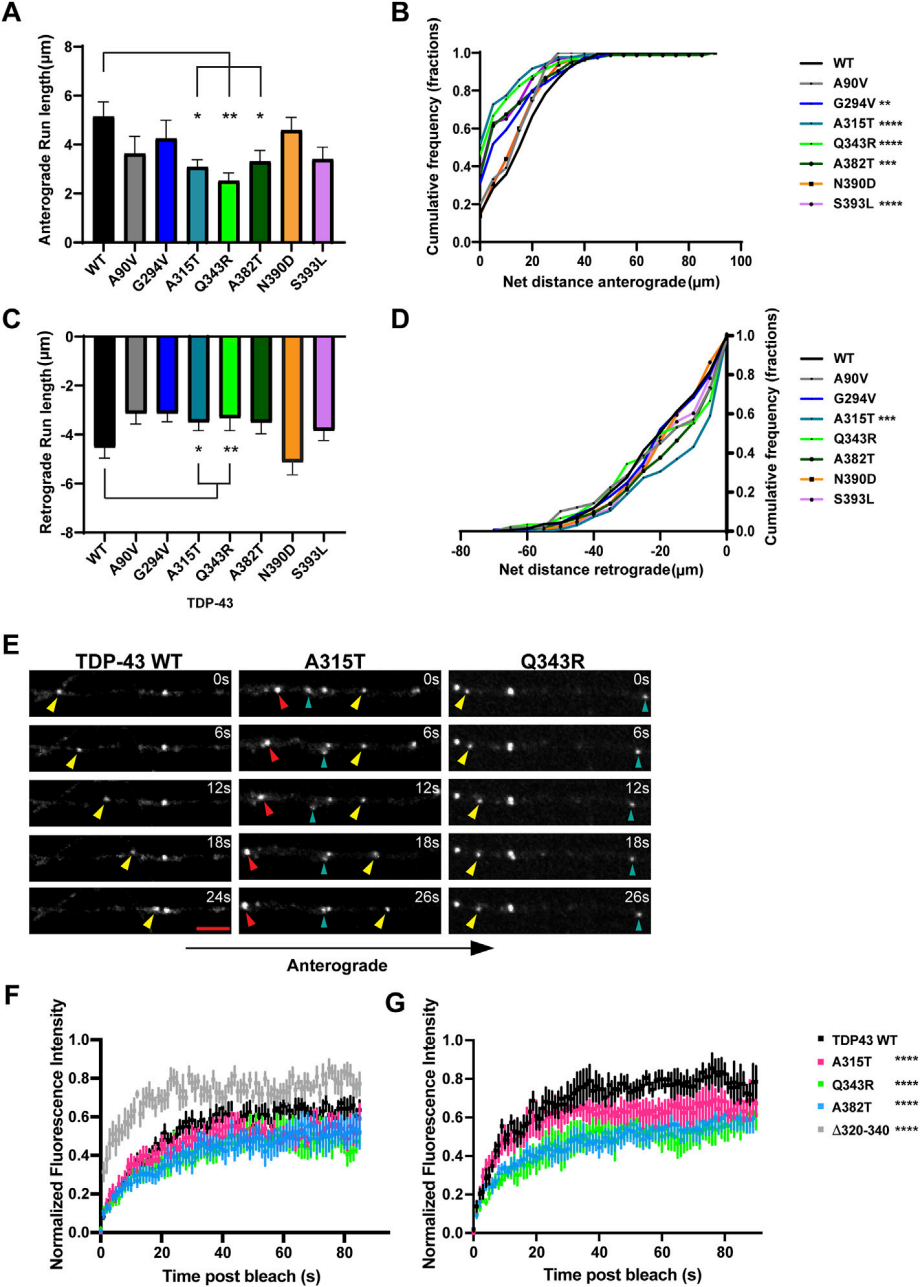
ALS-linked TDP-43 mutants A315T and Q343R exhibit defects in anterograde and retrograde transport. **(A)** Anterograde run lengths (μm) of WT or ALS-linked mutant TDP-43 RNP granules were determined using custom semi-automated analysis in MATLAB. Run lengths (mean ± SEM) are plotted (WT *n* = 63, G294V *n* = 54, A315T *n* = 85, Q343R *n* = 45, A382T *n* = 75, N390D *n* = 59, S393L, *n* = 64, and A90V *n* = 19, from 12 to 16 neurons for each condition, N = 3). **(B)** Cumulative frequency distribution of WT and ALS-linked TDP-43 RNP anterograde net distances from 3 independent experiments. **(C)** Retrograde run lengths (μm) of WT or ALS-linked mutant TDP-43 RNP granules were determined using custom semi-automated analysis in MATLAB; mean ± SEM is plotted (WT *n* = 100, G294V *n* = 77, A315T *n* = 125 and Q343R *n* = 65, A382T *n* = 85, N390D *n* = 45, S393L, *n* = 73, and A90V *n* = 26, from 12 to 16 neurons for each condition, N = 3). **(D)** Cumulative frequency distribution of WT and ALS-linked TDP-43 RNP retrograde net distances from 3 independent experiments. One way ANOVA (Kruskal–Wallis test with Dunn’s correction for multiple comparisons) was used to determine statistically significant difference among different samples, *p < 0.05, **p < 0.01, ***p < 0.0005 and ****p < 0.0001. **(E)** Time-lapse images of representative motile TDP-43 WT RNP granule (left panel, yellow arrowhead) travelling in the anterograde direction. Time-lapse images of representative ALS-linked TDP-43 mutants, A315T (middle panel) and Q343R (right panel), showing the most severe transport defects. A315T and Q343R RNP granules display reduced anterograde (yellow arrowhead) and/or retrograde (red arrowhead) runs. Oscillatory RNP granules are shown with cyan arrowheads; scale bar: 3 μm. **(F)** ALS-mutant RNP granules (A315T, *n* = 12; Q343R *n* = 8; A382T, *n* = 10 from N = 3 independent experiments) in the axon display reduced fluorescence recovery after whole bleach compared to TDP-43 WT RNP granules (*n* = 10); 2-way ANOVA with Tukey’s post-test for multiple comparisons, ****p < 0.0001. TDP-43 Δ320-330 RNP granules (*n* = 8) show more robust and rapid whole bleach recovery than WT (2-way ANOVA with Tukey’s post-test for multiple comparisons, ****p < 0.0001). **(G)** Fluorescence recovery after half-bleach of mid axonal TDP-43 WT granules (*n* = 9) or ALS-linked mutants [A315T (*n* = 9); Q343R (*n* = 5); A382T (n = 10)] from N = 3 independent experiments. TDP-43 WT RNP granules in the mid axon display more robust, rapid recovery after half-bleach, suggesting rapid internal molecular mobility reorganization within granules, compared to RNP granules containing A315T, Q343R, and A382T (2-way ANOVA with Tukey’s post-test for multiple comparisons, ****p < 0.0001). Normalized intensity values represent mean ± SEM.

Previous studies from our laboratory and others have shown that wild type TDP-43 RNP granules display bidirectional transport, with a slight bias in the anterograde direction (Alami et al., 2014; Gopal et al., 2017). In agreement with prior results (Fallini et al., 2012; Alami et al., 2014), we found that several ALS-linked TDP-43 mutants show defects in anterograde transport along the axon. Q343R and A382T mutants display a significantly reduced anterograde motile fraction, accompanied by an increased retrograde motile fraction in comparison to WT (Figure 1D). RNP granules containing A315T, Q343R, or A382T show significantly reduced anterograde run lengths (Figure 3A), while those containing N390D display significantly lower net anterograde velocity compared to WT RNP granules (Supplementary Figure S3A). In addition, all LCD ALS-mutants, except N390D, show significantly reduced net anterograde transport distance along the axon. Cumulative frequency distribution (CFD) of net anterograde transport distance shows that greater than 50% of TDP-43 WT RNP granules travel net distances of ≥15 μm, whereas less than ∼25% of RNP granules composed of G294V, A315T, Q343R, A382T, or S393L travel net distances of ≥15 μm (Figures 3B,E). In contrast, the A90V variant, which has been observed in healthy controls and in ALS patients, does not significantly alter anterograde run lengths, net anterograde distance, or velocity (Figures 3A,B, Supplementary Figure S3).

### TDP-43 A315T and Q343R Exhibit Altered Retrograde Transport and Biophysical Properties

Our data are consistent with prior studies showing that several ALS-linked TDP-43 mutations affect anterograde transport in the axon (Alami et al., 2014). TDP-43 knockdown, in contrast, significantly reduces both anterograde and retrograde RNP displacement and velocity in neurons (Chu et al., 2019). We asked whether any of the disease-associated mutations mimic defects observed with TDP-43 knockdown. We found that both mutations adjacent to the helical region, A315T and Q343R, significantly lowered retrograde run lengths compared to WT TDP-43 (Figures 3C,E). CFD of net transport distance shows that ∼50% of TDP-43 WT RNP granules travel distances further than 20 μm in the retrograde direction, whereas only 30% of RNP granules composed of A315T travel more than 20 μm net retrograde distance (*p* = 0.0009; Figure 3D). TDP-43 Q343R shows a similar trend in net retrograde distance, but this difference does not reach statistical significance. No other TDP-43 ALS-linked mutations that we examined showed consistent defects across multiple metrics of retrograde motility. For example, the net velocity of S393L granules travelling in retrograde direction is slightly increased compared to WT; however, the biological significance of this finding is unclear since retrograde run lengths and net distance are not altered. Similarly, the net retrograde velocity of motile N390D RNP granules trends downward compared to WT (*p* = 0.07) but run lengths and net distance of transport are preserved (Figure 3; Supplementary Figure S3). Taken together, these data suggest that axonal transport defects seen with TDP-43, A315T, and Q343R mutations affect both anterograde and retrograde motility of RNP granules and are more severe than those observed with other ALS-linked mutations.

Similar to our prior fluorescence recovery after photobleaching (FRAP) data (Gopal et al., 2017), we found that whole-bleach analysis of TDP-43 WT RNP granules demonstrates robust recovery of fluorescence intensity, suggesting that TDP-43 granules exist in dynamic equilibrium with the cytoplasmic pool of unbleached TDP-43. ALS-linked mutants TDP-43 A315T, Q343R, and A382T show slightly reduced dynamic exchange compared to WT, while TDP-43 Δ320-330 RNP granules display significantly more rapid recovery (*p* < 0.0001; Figure 3F). The half bleach data show slower and incomplete fluorescence recovery of ALS-linked mutant RNP granules compared to WT, suggesting reduced intramolecular mobility and altered biophysical properties within Q343R, A382T, and A315T RNP granules (*p* < 0.0001; Figure 3G).

### Specific Aromatic Residues and RGG Regulate Axonal Transport of TDP-43 RNP Granules

The collective interaction between associative motifs (“stickers”), such as tyrosine and arginine residues, drive phase separation of many intrinsically disordered proteins. These associative motifs are separated by “spacers” often containing polar residues, which modulate the material properties of condensates (Wang et al., 2018). In contrast, LLPS of TDP-43 largely depends on the conserved α-helical region with additional contributions from tryptophan residues, (G/S)-(F/Y)-(G/S) motifs, and a single RGG. Several studies have shown that disrupting cation-pi and pi-pi interactions reduce LLPS and partitioning into cellular condensates. Therefore, we asked whether substituting or replacing the aromatic or charged (arginine) residues also affect TDP-43 RNP condensate formation and axonal transport in neurons. Accordingly, we introduced phenylalanine or non-aromatic residues to replace single tryptophan residues (W334F/L, W385F/L and W412F/L) and tyrosine residue (Y374 F/T) (Supplementary Figure S1). Phenylalanine substitutions preserve the aromatic ring at these positions, whereas leucine substitutions eliminate the aromatic structure but have similar hydrophobicity as tryptophan. Likewise, substituting threonine for tyrosine confers a polar side chain but eliminates the aromatic ring. Since GFG and FG motifs participate in TDP-43 LLPS and are critical for the formation of TDP-43 fibrils (Pantoja-Uceda et al., 2021), we similarly replaced phenylalanine in GFG or FG/FS motifs with tyrosine or leucine (F276Y/L, F283Y/L, F289Y/L, F313L/Y, F313-316Y/L, F367Y/L, F397Y/L, and F401Y/L) (Supplementary Figure S1). We hypothesized substitutions that abolish the aromatic ring would show more severe effects on TDP-43 RNP condensate formation and transport compared to mutations that preserve the aromatic structure. In addition, we disrupted the only RGG found in the LCD of TDP-43 (R293 K/A), since there are five ALS-linked mutations associated with this motif (G294V, G294A, G295S, G295R, and G295C), highlighting the importance of this motif in TDP-43 biology (Phan et al., 2011).

**FIGURE 4:**
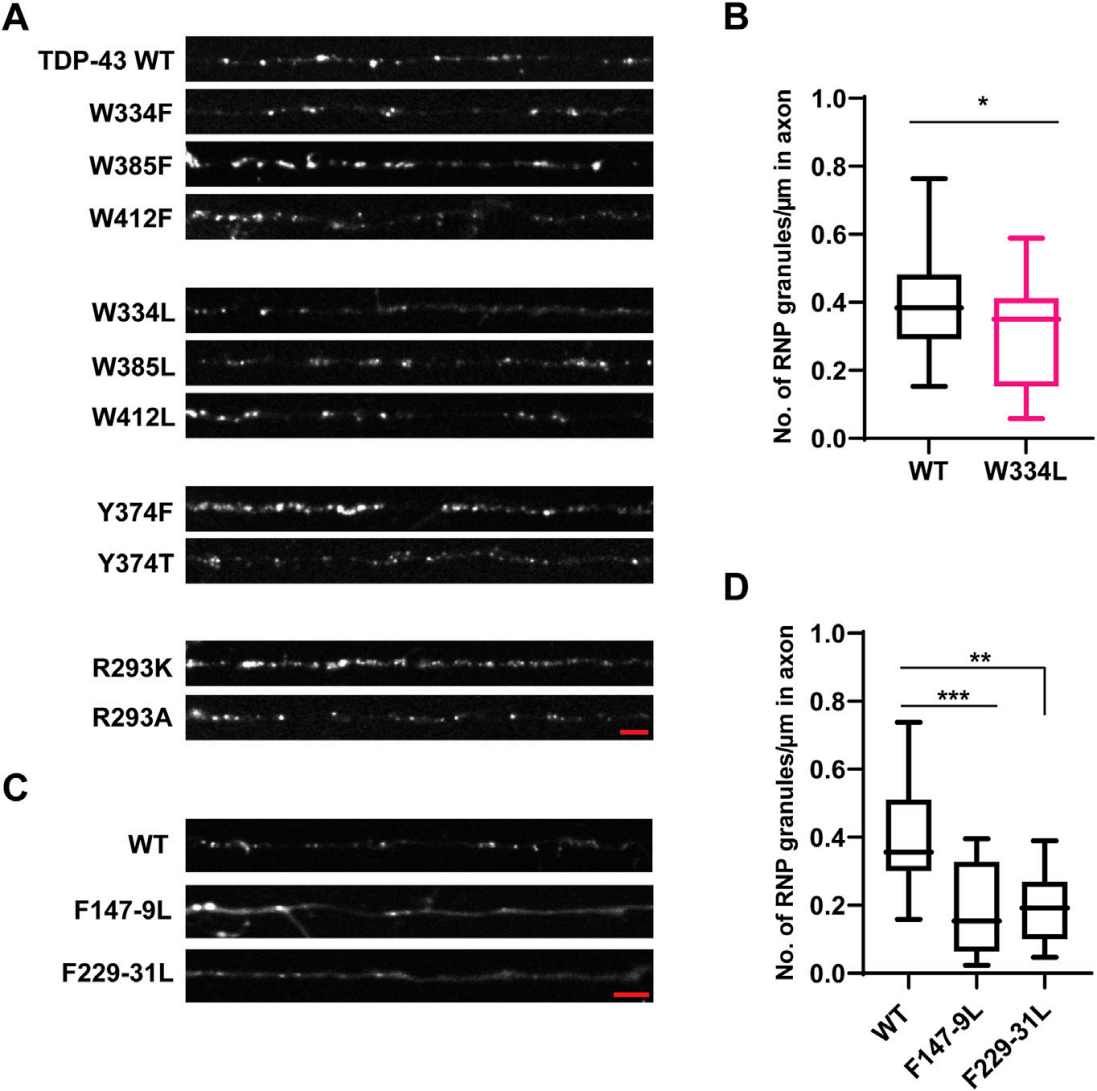
Aromatic mutant W334L affects RNP granule assembly. **(A)** TDP-43 RNP granules are observed along the axons of DIV 7–10 primary cortical neurons expressing the GFP tagged WT TDP-43, aromatic or RGG TDP-43 mutants. Representative images of WT or aromatic/RGG mutant RNP granules are shown. Scale bar (shown in red) corresponds to 3 μm. **(B)** Number of RNP granules per μm length of axon for WT TDP-43 and W334L TDP-43 mutants is plotted (see *Materials and Methods*) from *n* = 13–15 neurons per condition, from 3 independent experiments (*N* = 3). Error bars represent standard error mean. Unpaired *t-*test was used to determine statistically significant difference between WT and W334L TDP-43 RNP granules, *p < 0.05. **(C)** Representative images of GFP tagged WT TDP-43 and RRM mutant RNP granules are shown. Scale bar (shown in red) corresponds to 3 μm. **(D)** Number of RNP granules per μm length of axon for WT TDP-43 and TDP-43 RRM mutants is shown from *n* = 11–12 neurons per condition, from 3 independent experiments (*N* = 3). One way ANOVA with Dunn’s correction for multiple comparisons was used to test for statistically significant differences among samples; **p < 0.01, ***p < 0.001.

Prior studies have shown that three tryptophan residues (W334, W385, and W412) are critical for TDP-43 LLPS *in vitro*, with W334 in the conserved α-helical region having the greatest effect (Li et al., 2018b). Consistent with these results, we found that W334L and W412L variants form reduced numbers of TDP-43 positive RNP granules in the axon (W334L: 0.29 ± 0.04 granules/μm; W412L: 0.27 ± 0.03 granules/μm) compared to WT (0.40 ± 0.03 granules/μm, *p* < 0.05; Figures 4A,B; Supplementary Figures S4A,B). However, these reductions are milder than those seen with TDP-43 Δ320-330 (compare Figure 1B). Substitutions which preserve the aromatic ring at these positions (W334F, W412F) and W385F/L variants do not affect the number of neuronal TDP-43 RNP granules in the axon (Figure 4A, Supplementary Figures S4A, B).

**FIGURE 5:**
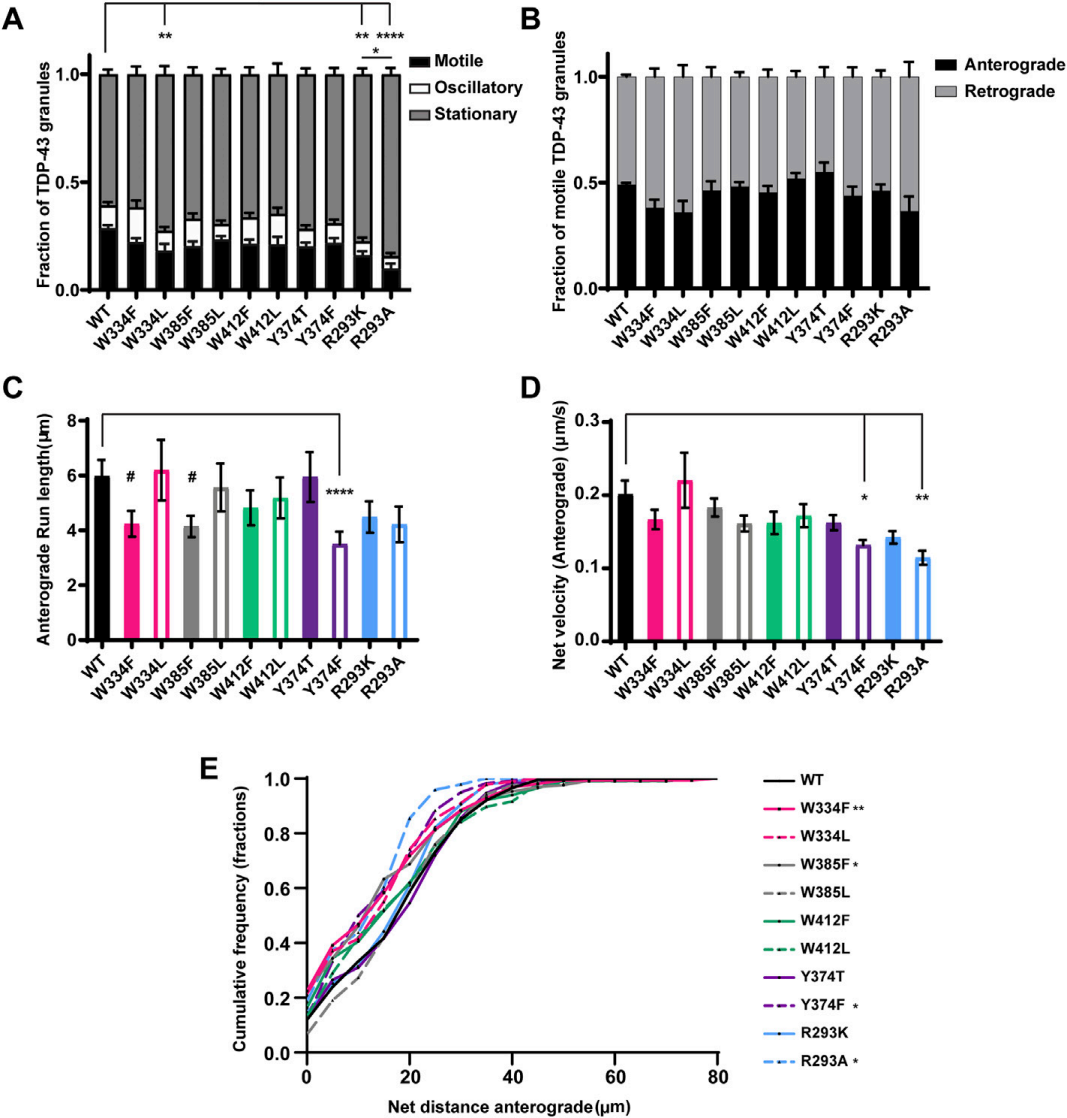
TDP-43 mutations disrupting RGG motif of LCD and W residue of α-helical domain display defects in anterograde transport. **(A)** WT TDP-43 or TDP-43 LCD W/Y/R mutant kymographs from 3 independent experiments are analyzed to determine the motility of different RNP granules. Fraction of motile, oscillatory and stationary RNP granules are plotted. **(B)** The fraction of motile RNP granules for WT TDP-43 and each of the TDP-43 LCD W/Y/R mutant (shown as black bars in Figure 5A), are further categorized as granules travelling in retrograde or anterograde direction and the resulting fraction is plotted. **(C)** The run lengths (μm) of motile WT TDP-43 or TDP-43 LCD W/Y/R mutant RNP granules moving in anterograde direction are determined using custom semi-automated analysis in MATLAB software and the resulting run lengths are plotted (N = 3, WT *n* = 75, W334F *n* = 72, W385F *n* = 87 and Y374F *n* = 80 from 13 to 15 neurons from each condition). **(D)** The anterograde net velocity (μm/s) for each of the motile WT TDP-43 or TDP-43 LCD W/Y/R mutant RNP granule was determined using custom semi-automated analysis in MATLAB and the average net velocity is plotted (N = 3, WT *n* = 88, Y374F *n* = 105 and R293A *n* = 40). Error bars represent standard error mean. **(E)** Cumulative frequency distribution of TDP-43 WT and LCD W/Y/R mutant RNP anterograde net distances, N = 3 independent experiments. One way ANOVA (Kruskal–Wallis test with Dunn’s correction for multiple comparisons) is used to determine the statistically significant difference among different samples. #*p* < 0.1, *p < 0.05, **p < 0.01, ***p < 0.0005 and ****p < 0.0001.

Apart from their contribution to LLPS, little is known about the functional role of tryptophan residues in the LCD of TDP-43. Out of the three tryptophan residues, one ALS-associated mutation W385G, has been described (Buratti, 2015), and a prior study suggested that W385 and W412 contribute to TDP-43 splicing function, independent of their role in LLPS (Schmidt et al., 2019). Using published Consurf and conseq tools, we analyzed conservation and predicted structurally and functionally important residues in TDP-43 based on sequences from 47 eukaryotic species (Supplementary Table S2). We found that all three tryptophan residues show a high degree of conservation, and W412 is predicted to be an exposed, functional residue (Supplementary Figures S5, S6). Therefore, we sought to determine the functional role of each W residue in TDP-43 RNP axonal transport, using the same experimental approach as described for the ALS-linked mutants. Given the high degree of conservation at W334, W385, and W412 and because tryptophan is the only amino acid which contains an indole ring, we hypothesized that substitutions of W with either F or L could compromise TDP-43 RNP transport. We found that W334L substitution, but not W to F substitution (W334F), reduces the motile fraction of RNP granules containing this TDP-43 variant (W334L motile fraction 0.18 ± 0.03) compared to WT (0.28 ± 0.01; *p* < 0.01) (Figure 5A). Neither F or L substitutions at W385 and W412 significantly changed the fraction of motile RNP granules. These data may suggest TDP-43 W334L is less efficiently recruited to neuronal RNP condensates containing functional components required for motility.

**FIGURE 6:**
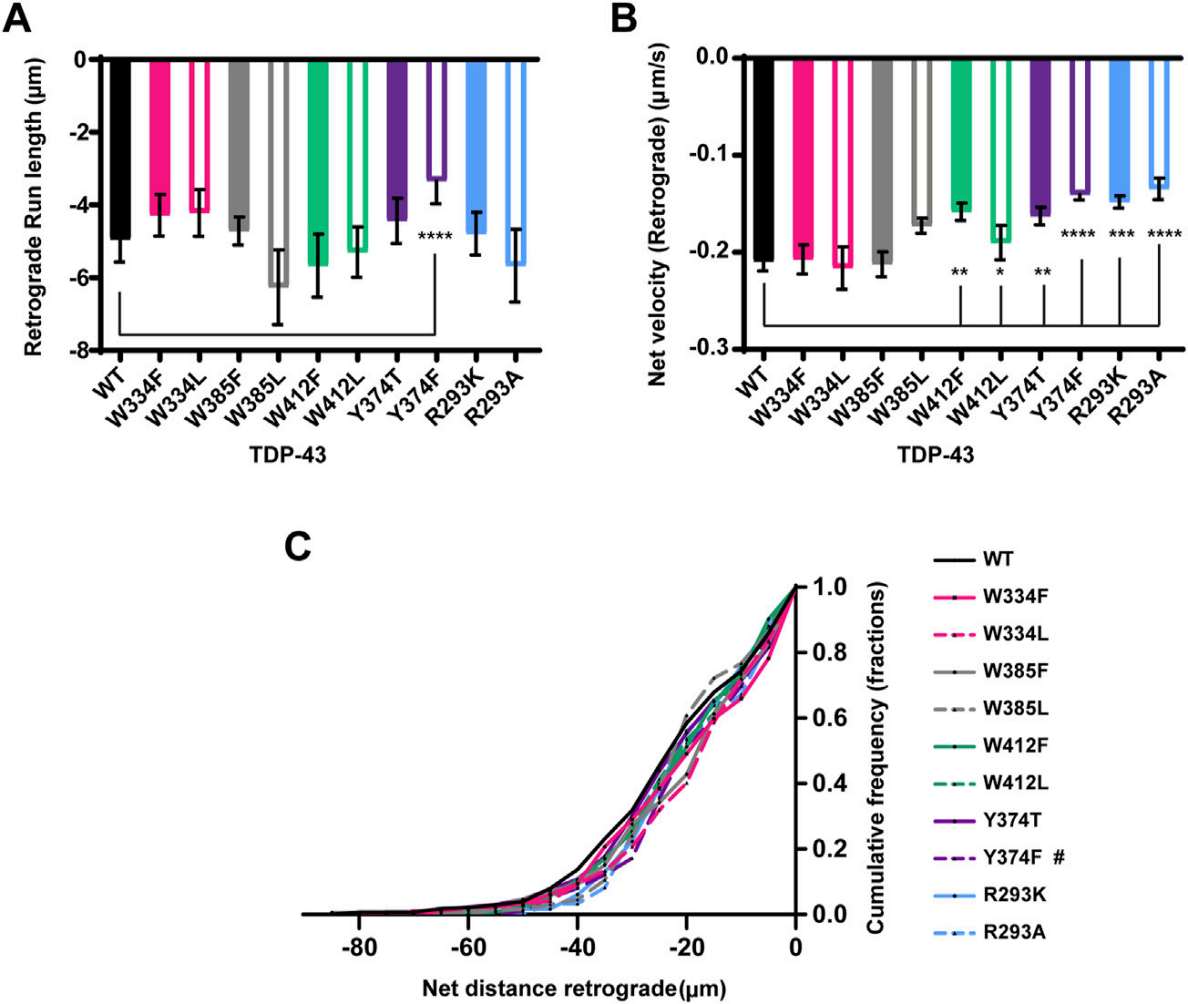
TDP-43 mutations disrupting Y374 in the LCD may affect retrograde motility. **(A)** The run lengths (μm) of motile WT TDP-43 or TDP-43 LCD W/Y/R mutant RNP granules moving in retrograde direction are determined using custom semi-automated analysis in MATLAB and the resulting run lengths are plotted (N = 3, WT *n* = 38 and Y374F *n* = 89). **(B)** The retrograde net velocity (μm/s) for each of the motile WT TDP-43 or TDP-43 LCD W/Y/R mutant RNP granule was determined using MATLAB software and the average net velocity is plotted (N = 3, WT *n* = 120, W412F *n* = 86, W412L *n* = 82, Y374T *n* = 91, Y374F *n* = 114, R293K *n* = 82, and R293A *n* = 41). Error bars represent standard error mean. **(C)** Retrograde net distance cumulative frequency distribution of TDP-43 WT and LCD W/Y/R mutant RNP granules, N = 3 independent experiments. One way ANOVA (Kruskal–Wallis test with Dunn’s correction for multiple comparisons) was used to determine the statistically significant difference among different samples. #*p* < 0.1, *p < 0.05, **p < 0.01, ***p < 0.0005, and ****p < 0.0001.

None of the W substitutions significantly affected the fraction of anterograde versus retrograde motile tracks, anterograde net velocity, or anterograde run lengths, although W334F and W385F mutants show a trend towards reduced anterograde run length (#*p* = 0.08; Figures 5B–D). CFD of net anterograde transport distance shows that ∼55% of TDP-43 WT RNP granules travel net distances of ≥15 μm, whereas only ∼40% of RNP granules composed of W334F and W385F travel net distances of ≥15 μm (W334F *p* < 0.01, W385F *p* < 0.05; Figure 5E). Similarly, ∼45% of RNP granules containing W334L travel net distances of ≥15 μm, but this reduction did not reach statistical significance (*p* = 0.12; Figure 5E). Interestingly, substitutions at W412, which is predicted to be an exposed functional residue, significantly reduce the net retrograde velocity, but not retrograde run lengths, of motile RNP granules (Figures 6A,B). However, the biological significance of this defect is unclear since net retrograde distance is not affected for either W412F or W412L (Figure 6C). Taken together, our data suggest an aromatic residue at position 334 is important for efficient recruitment to motile RNP condensates in neurons (Figure 5A). However, phenylalanine cannot substitute functionally for tryptophan with regard to specific aspects of TDP-43 anterograde and retrograde transport; the consequences of these substitutions are mild though, compared to transport defects seen with ALS-linked TDP-43 mutations.

Interactions between tyrosine and arginine residues are critical for FUS phase separation (Wang et al., 2018); however, *in vitro* data suggest that arginine-mediated electrostatic interactions are not essential for TDP-43 LLPS and disrupting Y374 does not significantly impair LLPS either (Li et al., 2018b; Schmidt et al., 2019). To clarify the role of arginine and RGG motif at residues 293–295 in TDP-43 RNP condensate formation, R293 was replaced either with lysine, preserving the positive charge (R293K), or alanine (R293A). In addition, to determine whether disruption of the RGG, a known RNA-binding motif in FMRP (Phan et al., 2011) and other RNA-binding proteins (Thandapani et al., 2013) has similar effects as disruption of the RRM, we examined TDP-43 RNP condensate formation of RRM1 (F147L-149L) and RRM2 RNA-binding mutants (F147-9L and F229-31L) (Buratti and Baralle, 2001; Lukavsky et al., 2013). Similarly, we asked whether replacing tyrosine with a hydrophobic aromatic residue (Y374F), or a polar non-aromatic residue (Y374T), affects formation of TDP-43 RNP condensates in neurons. We found that neither substitutions at R293 K/A or Y374 F/T significantly affect the average number of TDP-43 positive RNP granules in the axon (Figure 4A; Supplementary Figure S4A,C). However, simple linear regression analysis demonstrates a steeper positive correlation between the number of Y374F positive granules per micron as a function of axonal fluorescence intensity (proxy for expression level) (Y374F slope = 0.028 ± 0.004), compared to TDP-43 WT (WT slope = 0.015 ± 0.003, *p* < 0.02; Supplementary Figure S4C). These data suggest a more sensitive assay may be needed to determine if Y374F enhances partitioning of TDP-43 into neuronal RNP condensates.

Prior studies have shown that the addition of RNA facilitates LLPS of purified TDP-43 CTD *in vitro* (Conicella et al., 2016). Full-length TDP-43 RNA binding mutants antagonize aberrant phase transitions (Mann et al., 2019), and binding of specific RNA sequences increase the liquid properties of TDP-43 condensates (Grese et al., 2021). In agreement with these studies, we observed that mutations of either RRM1 (F147-149L) or RRM2 (F229-231L) significantly reduced the recruitment of TDP-43 to RNP granules and/or formation of TDP-43 RNP condensates in neurons (F147-149L: 0.19 ± 0.04 granules/μm and F229-231L: 0.20 ± 0.03 granules/μm) compared to WT TDP-43 (0.40 ± 0.04; *p* ≤ 0.0015; Figures 4C,D).

The importance of the RGG and single tyrosine (Y374) on TDP-43 RNP transport is not known, but the existence of several ALS-associated mutations in the vicinity of R293 and a nonsense mutation (Y374X) that produces a truncated form of TDP-43 (TDP-43 1–373) raise the possibility of a functional role (Buratti, 2015). Conservation analysis and prediction of functional residues indicate that both Y374 and R293 are highly conserved and predicted to be exposed, functional residues (Supplementary Figures S5, S6). We found that disrupting RGG with either R293K or R293A substitutions profoundly reduces TDP-43 RNP granule motility (Figure 5A). The motile fraction of TDP-43 R293A (0.09 ± 0.02) or R293K (0.16 ± 0.01) is significantly lower than the motile fraction of TDP-43 WT (0.28 ± 0.01; *p* < 0.001) (Figure 5A). Moreover, R293A motility is significantly reduced compared to R293K (*p* = 0.02), suggesting that a positive charge at this residue is critical for long-range motility and/or interactions with motors/adaptors. Consistent with this notion, RNP granules containing R293A exhibit significantly reduced anterograde net velocity and net transport distance compared to WT (*p* < 0.01; Figures 5D,E). The CFD of anterograde transport shows that ∼40% of R293A RNP granules travel net distances ≥15 μm and only 15% of R293A RNP granules travel net distances ≥20 μm; by comparison, ∼55% of WT RNP granules travel net distances ≥15 μm and greater than 40% of WT RNP granules travel net distances ≥20 μm (*p* < 0.05, Figure 5E). RNP granules containing R293A or R293K also show defects in retrograde motility, but these are milder than the anterograde deficits. Retrograde run lengths are preserved, but both R293K and R293A display a significant reduction in retrograde net velocity compared to WT (R293A *p* < 0.0001 and R293K *p* < 0.0005; Figures 6A,B). There is no significant change in the CFD of retrograde net distance for either R293K or R293A variants, however. These data highlight the importance of the positively charged arginine residue in regulating multiple aspects of TDP-43 RNP motility, as seen from the severe phenotypes observed with R293A substitution.

Next, we used Y374 F/T substitutions to determine whether Y374 also plays a role in TDP-43 RNP granule transport. Neither of these substitutions alter the fraction of motile RNP granules, or the fraction of anterograde vs. retrograde motility (Figures 5A,B). Interestingly, Y374F substitutions profoundly reduce anterograde and retrograde run lengths (*p* < 0.0001) and net velocity (*p* < 0.05) compared to WT RNP granules (Figures 5C,D and Figures 6A,B). Consistent with these defects, the anterograde net distance CFD for Y374F RNP granules is left-shifted relative to the WT CFD. Only ∼40% of Y374F RNP granules transport net distances of ≥15 μm, whereas ∼55% of WT granules travel the same net distance (*p* < 0.05, Kruskal–Wallis test). In contrast, Y374T substitution has no effect on any aspect of anterograde motility (Figure 5) and only shows a mild reduction in retrograde net velocity compared to WT (*p* < 0.01; Figure 6B) such that net distance of retrograde transport is not affected (Figure 6C). Taken together, these results suggest the polar side chain of the amino acid at position 374 is more critical than the aromatic ring structure, as substitution of a polar aromatic (Y) to a hydrophobic aromatic (F) severely hindered axonal transport of TDP-43.

### Role of RNA Binding in TDP-43 RNP Granule Transport

**FIGURE 7:**
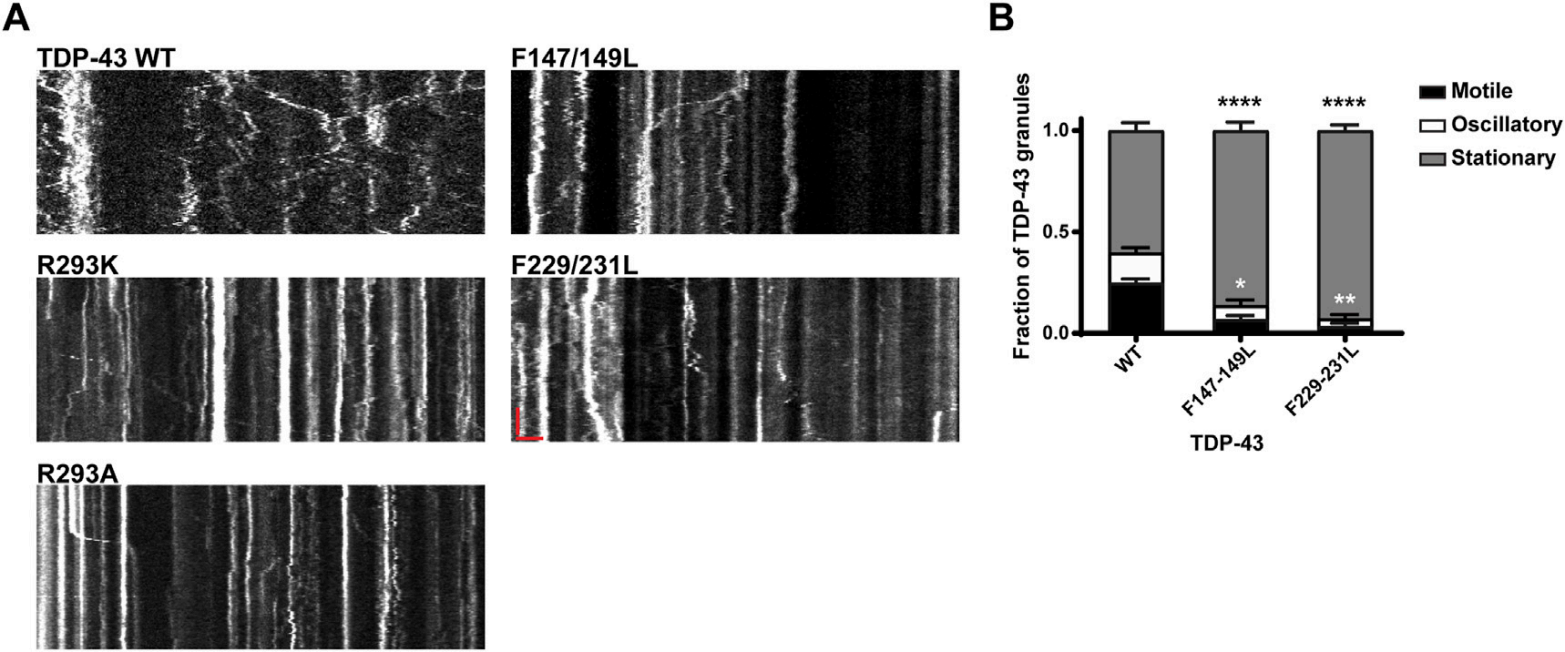
Neuronal RNP granules composed of TDP-43 RRM1/RRM2 mutants exhibit reduced motility. **(A)** Representative kymographs of GFP tagged TDP-43 WT, RGG mutant, or RRM1/2 mutant RNP granules are shown. In the kymographs, the retrograde direction (towards the Soma) is to the left and the anterograde direction is to the right. The scale bar (horizontal red line) corresponds to 3 μm (*x* axis) and time scale (vertical red line) corresponds to 50 s (*y* axis). **(B)** Fraction of TDP-43 WT or RRM1/2 mutant RNP granules that are stationary, oscillatory, or motile in the axon; motile puncta ≥10 μm net displacement in 5 min. TDP-43 RRM1 (F147-149L) and RRM2 (F229-231L) mutants exhibit significantly reduced motile (black asterisk) and oscillatory fraction (white asterisk), compared to WT. For each condition, *n* = 11–12 neurons were analyzed from 3 independent experiments (*N* = 3). Error bars represent standard error mean. One way ANOVA with Dunnett’s correction for multiple comparisons was used to determine statistically significant differences among samples; *p < 0.05, **p < 0.01, and ****p < 0.0001.

Our results showing markedly reduced RNP motility in R293K/A mutants raised the possibility that the RGG is crucial for TDP-43 interaction with specific mRNAs, RNA-dependent interactions with other RNA-binding proteins and/or motor proteins, including kinesin and the cytoplasmic dynein/dynactin complex. To test this hypothesis fully, multiple approaches will be needed, but as a first step towards understanding the role of RNA in regulating neuronal RNP transport, we studied TDP-43 RNP transport in neurons expressing RRM mutants (F147-149L or F229-231L). We observed that RRM1/2 mutants exhibit significantly reduced long-range oscillatory motility, similar but more severe than RGG mutants (Figure 5A; Figures 7A,B). Compared to the motile fraction of TDP-43 WT RNP granules (0.25 ± 0.02), both RRM1 (F147-149L) and RRM2 mutant (F229-231L) exhibit significantly reduced motile fraction (F147-149L: 0.07 ± 0.02; F229-231L: 0.03 ± 0.01; *p* < 0.0001). These results suggest that RNA binding is important for TDP-43 assembly into RNP condensates (Figures 4C,D) and is also a critical determinant of RNP granule motility (Figure 7).

### Conserved Phenylalanine Residues in LARKS Regulate Axonal Transport of TDP-43 RNP Granules

**FIGURE 8:**
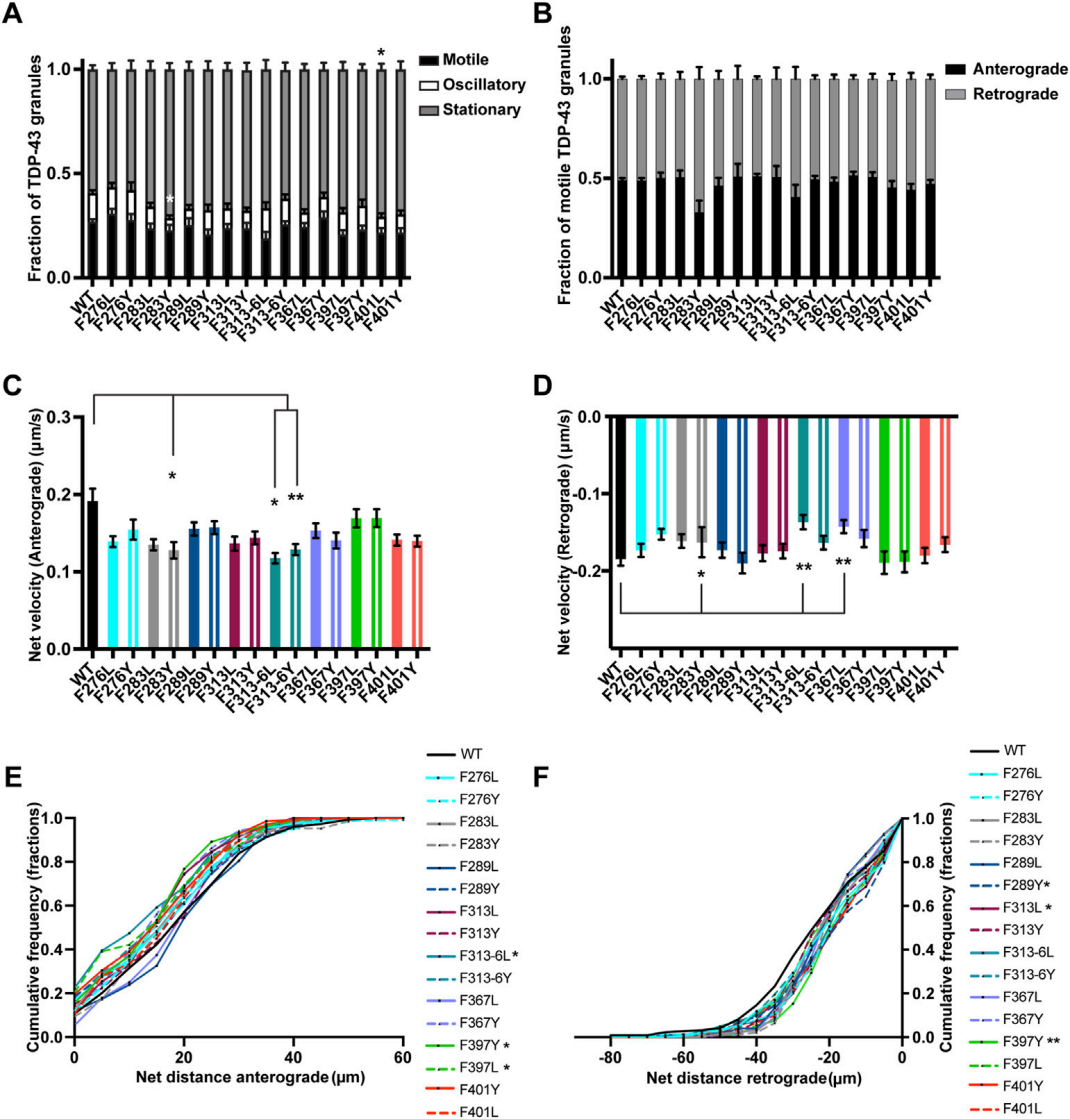
TDP-43 mutation disrupting F in LARKS affects RNP transport. **(A)** WT TDP-43 or TDP-43 LCD F mutant kymographs from 3 independent experiments are analyzed to determine the motility of different RNP granules. Fraction of motile, oscillatory and stationary RNP granules are plotted. **(B)** The fraction of motile RNP granules for WT TDP-43 and each of the TDP-43 LCD F mutant (shown as black bars in Figure 8A), are further categorized as granules travelling in retrograde or anterograde direction and the resulting fraction is plotted. **(C)** The anterograde net velocity (μm/s) for each of the motile WT TDP-43 or TDP-43 LCD F mutant RNP granule was determined using custom semi-automated analysis in MATLAB and the average net velocity is plotted (N = 3, WT *n* = 104, F276L *n* = 105, F276Y *n* = 98, F283L *n* = 88, F283Y *n* = 49, F289L *n* = 72, F289Y *n* = 71, F313L *n* = 85, F313Y *n* = 101, F313-6L *n* = 43, F313-6Y *n* = 124, F367L *n* = 86, F367Y *n* = 95, F397L *n* = 75, F397Y *n* = 84, F401L *n* = 71, F401Y *n* = 91, from 9 to 15 neurons per condition). **(D)** The retrograde net velocity (μm/s) for each of the motile WT TDP-43 or TDP-43 LCD F mutant RNP granule was determined using custom semi-automated analysis in MATLAB and the average net velocity is plotted (N = 3, WT *n* = 128, F276L *n* = 109, F276Y *n* = 125, F283L *n* = 95, F283Y *n* = 75, F289L *n* = 79, F289Y *n* = 70, F313L *n* = 82, F313Y *n* = 100, F313-6L *n* = 59, F313-6Y *n* = 124, F367L *n* = 92, F367Y *n* = 90, F397L *n* = 73, F397Y *n* = 100, F401L *n* = 88, F401Y *n* = 99). Error bars represent standard error mean. **(E)** Anterograde or **(F)** retrograde net distance cumulative frequency distribution for TDP-43 WT or LCD F mutant RNP granules, from 3 independent experiments. One way ANOVA (Kruskal–Wallis test with Dunn’s correction for multiple comparisons) was used to determine statistically significant difference among different samples. *p < 0.05, **p < 0.01.

There are five phenylalanine residues (F276, F283, F289, F313, and F316) in IDR1 (274–316) preceding the conserved α-helical region. Of these residues, F283 and F289 form GFG LLPS motifs, while F313 and F316 contribute to a LARKS (Li et al., 2018b; Guenther et al., 2018; Schmidt et al., 2019). We were interested to define the role of these phenylalanine residues in TDP-43 RNP granule transport. Prior studies have demonstrated that mutating or deleting GFG and FG LLPS motifs does not affect phase separation of purified TDP-43 but instead impacts TDP-43 fibrillization (Li et al., 2018b; Pantoja-Uceda et al., 2021). In addition, F to Y substitutions do not alter TDP-43 dynamics as measured by half bleach fluorescence recovery (Schmidt et al., 2019). In neurons we found that individual F to L or F to Y substitutions do not significantly affect the average number of TDP-43 RNP granules per micron (Supplementary Figure S7A). Likewise, single substitutions of phenylalanine residues in IDR1 do not affect the fraction of motile granules or ratio of anterograde to retrograde motility (Figures 8A,B). However, substituting a tyrosine for a conserved phenylalanine F283Y, results in a significant loss of oscillatory motility (F283Y oscillatory fraction 0.05 ± 0.01 vs. WT 0.14 ± 0.01; *p* < 0.01; Figure 8A) and reduces the net velocity in both anterograde and retrograde directions compared to WT TDP-43 (*p* < 0.05; Figures 8C,D). In contrast, the F283L substitution, which disrupts the GFG LLPS motif, does not cause any motility defects at all. Substitutions of a less conserved phenylalanine, F289Y/L, do not alter motility or net velocities, though F289Y (but not F289L) displays a small but statistically significant defect in net distance of retrograde transport and a trend towards reduced retrograde run length compared to the WT (Figure 8F; Supplementary Figures S6, S7C). These results suggest the hydrophobicity of the phenylalanines in this region (residues 277–301) may be more important for TDP-43 transport than the aromatic structure.

We went on to test whether substitutions of one or both of the highly conserved F313 and F316 residues in the LARKS affect TDP-43 RNP transport (Supplementary Figures S5, S6) (Guenther et al., 2018). Because of their proximity to the α-helical region and structural contribution to LARKS, we hypothesized that mutating one or both phenylalanine residues would cause defects in TDP-43 transport. Interestingly, loss of the aromatic ring with the F313L mutant leads to a defect in the net distance of retrograde transport; the retrograde net distance CFD shows that only ∼34% of RNP granules containing F313L travel net distances ≥25 μm, whereas ∼50% of TDP-43 WT RNP granules travel the same distance (*p* < 0.05; Figure 8F). Transport defects are not seen when the aromatic ring is preserved (F313Y). Mutating both F313 and F316 results in a more severe phenotype, with the F313-6L substitution showing worse transport deficits than F313-6Y. F313-6L mutant exhibits reduced anterograde and retrograde net velocities (Figures 8C,D), and this was accompanied by a significant reduction in net distance of anterograde transport (Figure 8E). The anterograde net distance shows that only ∼32% of RNP granules containing F313-6L transport net distances ≥15 μm, whereas ∼55% of TDP-43 WT RNP granules travel the same distance (*p* < 0.05; Figure 8E). F313-6Y mutant, which preserves the aromatic rings at these residues, only exhibits lower anterograde net velocity (Figure 8C) and does not affect net distance of transport (Figures 8E,F). These data are quite different than the results seen above for F283, for example, and suggest the conserved aromatic residues in LARKS play an important structural role in TDP-43 RNP granule transport.

There are 2 GF/GFG motifs and one FG motif in IDR2 region (346–414) (Supplementary Figure S1). Since an ALS-linked mutation (G368S) has been described (Chio et al., 2012) that alters the FG motif at F367, we substituted Y or L (F367Y/L) at this residue. We found that loss of aromatic residue at this position (F367L) slows net velocity of retrograde transport (*p* < 0.01; Figure 8D), but there is no defect in net transport in the retrograde direction, suggesting this substitution has a mild effect (Figure 8F). In contrast, F to Y substitutions do not impact any aspect of TDP-43 RNP transport, suggesting that tyrosine’s aromatic ring at this position is able to compensate for phenylalanine and maintain TDP-43 transport function (Figures 8A–F).

Disruption of the GF/GFG motifs present in IDR2 with F397Y/L or F401Y/L substitutions does not affect the fraction of motile and oscillatory TDP-43 RNP granules, but there is a small increase in the fraction of stationary F401L RNP granules compared to WT (*p* < 0.05; Figure 8A). F401Y/L substitutions, however, do not impact any aspect of anterograde or retrograde net velocity or transport of TDP-43 RNP granules (Figures 8B–F). In contrast, both leucine and tyrosine substitutions at F397 significantly skew the CFD of anterograde net distance to the left compared to TDP-43 WT granules (*p* < 0.05; Figure 8E). Further, F397Y also showed significantly reduced retrograde transport as seen from the retrograde net distance CFD, where only ∼30% of F397Y RNP granules travel distance of ≥25 μm but ∼50% of WT RNP granules travel the same distance (*p* > 0.01; Figure 8F). Taken together, these results suggest that the highly conserved GF motif at F397 in IDR2 (Supplementary Figure S6) is important for maintaining anterograde and retrograde net transport distance. Tyrosine, despite being an aromatic, is unable to compensate functionally, suggesting a hydrophobic phenylalanine residue may be needed.

## Discussion

TDP-43 and other ALS-linked RNA-binding proteins are critical components of cellular biomolecular condensates, specifically ribonucleoprotein (RNP) granules, which regulate post-transcriptional processing of RNA, and mRNA localization and translation (Banani et al., 2017). Purified RNA-binding proteins including TDP-43, and cellular RNP granules display liquid-like biophysical properties, consistent with the notion that they assemble through LLPS (Brangwynne et al., 2009). Grasping the functional implications of TDP-43 phase separation *in vivo* and of aberrant phase transitions in disease, are essential for understanding ALS pathophysiology. Recent exciting work suggest the conserved α-helical region fine-tunes TDP-43 phase separation and modulates its RNA-binding and splicing function as well as translation *in vivo* (Conicella et al., 2020; Gao et al., 2021; Hallegger et al., 2021). Additional studies are needed however, since the α-helical region also mediates heterotypic interactions with other RNA-binding proteins (D'Ambrogio et al., 2009).

Multiple elegant studies have shown that ALS-associated mutations promote aberrant liquid to solid phase transitions of several RNA-binding proteins (Molliex et al., 2015; Patel et al., 2015; Conicella et al., 2016; McGurk et al., 2018). Data on the functional consequences of liquid to solid transitions in neurons is more limited, however. We and others have shown ALS-linked mutations impair TDP-43 mobility in axons (Alami et al., 2014; Gopal et al., 2017) and dendrites (Liu-Yesucevitz et al., 2014) and reduce net anterograde transport of *Nefl* mRNA in the axon (Alami et al., 2014). Moreover, we have shown that TDP-43 ALS-linked mutations arrest molecular mobility within RNP granules, perturbing the dynamic liquid-like properties of RNP transport granules in the axon (Gopal et al., 2017). The molecular mechanisms underlying mutant TDP-43 RNP transport defects remain unclear, however. In fact, the structural elements and/or amino acid sequence determinants that underlie TDP-43 RNP transport and interactions with motors and adaptors are not well-understood. Therefore, in this study we compared the functional impact of TDP-43 ALS-linked LCD mutations located within IDR1, IDR2, or near the conserved helical region on TDP-43 RNP transport, and we performed a mutagenesis study of structural elements, aromatic, and charged residues in the LCD to identify key determinants of TDP-43 RNP transport in neurons.

Evidence suggests anterograde transport of mRNA in neuronal RNP granules may be directly mediated by kinesin-1 (KIF5) and/or kinesin-2 (KIF3A/B and KAP3), while retrograde transport toward the cell body is driven by cytoplasmic dynein, its activator dynactin, and cargo-specific activating adaptors (Kanai et al., 2004; Bullock et al., 2006; Baumann et al., 2020). Longer run lengths and faster velocities can be explained by the ability of cargo-specific activating adaptors to recruit two or more motors to each transport complex (Guedes-Dias and Holzbaur, 2019; Baumann et al., 2020). Although certain RNA-binding proteins are necessary for active transport of mRNA, few have been shown to interact directly with motor proteins and are likely to bind as complexes of RNA-binding proteins, motors, and adaptors (Kanai et al., 2004; Elvira et al., 2006; Dictenberg et al., 2008). Phase separation offers a possible mechanism to assemble complexes of RNA-binding proteins with motors/adaptors directly, or through interactions (“hitchhiking”) with membrane-bound organelles (Buxbaum et al., 2015; Liao et al., 2019). In support of this notion, interactions between TDP-43 and FMRP may facilitate assembly with kinesin-1, whereas interaction with Staufen1 may mediate association with dynein (Chu et al., 2019). Interestingly, we found that TDP-43 mutations previously shown to disrupt phase separation *in vitro*, such as deletions of the conserved α-helical domain (Δ320-330, Δ320-340) (Conicella et al., 2016) and mutations of W334 (Li et al., 2018b), significantly reduce long-range, directed motility (net distance ≥10 μm) of RNP granules (Δ320-330 motile fraction, Figure 1C; W334L motile fraction, Figure 5A). In addition, Δ320-330 RNP granules exhibit defects in anterograde run lengths (Figure 2B), suggesting that TDP-43 α-helical domain mutants are less efficiently recruited to neuronal RNP condensates containing motors or adaptor proteins, particularly those required for anterograde motility (Amrute-Nayak and Bullock, 2012; Buxbaum et al., 2015). A non-mutually exclusive possibility is that these mutations may reduce assembly with late endosomes/lysosomes and impair hitchhiking on membrane-bound organelles.

Consistent with prior studies (Alami et al., 2014; Gopal et al., 2017), we found that all ALS-associated TDP-43 mutations cause defects in anterograde transport, to varying degrees. Furthermore, we observed that A315T and Q343R mutations adjacent to the α-helical domain show the most severe defects in anterograde transport as well as perturbations of retrograde transport (Figures 1A,C; Figure 3). A315 and Q343 residues both participate in minor populations of short helical conformations (Conicella et al., 2016). In addition, A315 is within the LARKS (residues 312–317) that forms labile kinked β-sheets, while Q343 is part of a steric zipper structure (residues 333–343) (Guenther et al., 2018). Moreover, A315T mutation has been reported to strengthen LARKS, promoting conversion to irreversible aggregates *in vitro* (Guenther et al., 2018). Indeed, our FRAP data supports loss of dynamic, liquid-like properties in RNP granules containing several ALS-linked TDP-43 mutants [(Gopal et al., 2017); Figures 3F,G]. Therefore, we speculate that A315T and Q343R mutations may perturb biophysical properties of TDP-43 RNP granules and/or lead to structural alterations that prevent association with 1) motors, directly or indirectly (e.g., by reducing association with FMRP); 2) activating adaptors needed for transport [e.g. KAP3 (Baumann et al., 2020),] or 3) adaptors needed to link RNP condensates to late endosomes and lysosomes [e.g. Annexin A11 (Liao et al., 2019)].

Similarly, substitutions of conserved aromatic residues within the conserved α-helical domain (W334F/L) or LARKS (F313L, F313-6L, and F313-6Y) exhibit defects in TDP-43 RNP granule transport. Anterograde transport appears to be more sensitive to disruptions of these structural elements than retrograde transport, as mutations of either the α-helical domain or LARKS impair anterograde run lengths, net velocity, and/or net distance (Figure 2B, Figures 5C–E; Figures 8C,E). In contrast, defects in retrograde velocity and/or net distance are seen with mutations of LARKS (Figures 8D,F), but not with deletions or substitutions in the α-helical domain (Figure 6). These data suggest conserved structural elements are important determinants of TDP-43 RNP granule transport and may reflect less efficient binding to molecular motors and/or adaptor proteins.

The phase separation behavior of TDP-43 depends on net charge of the protein as well as availability of RNA (Conicella et al., 2016; Li et al., 2018a). Increased concentrations of RNA can drive phase separation of TDP-43 and inhibit aberrant phase transitions (Mann et al., 2019; Pakravan et al., 2021). Furthermore, an increasing number of mRNA localization elements proportionately increases recruitment of motors and adaptors to an RNP granule (Amrute-Nayak and Bullock, 2012), suggesting that disrupting the RGG in TDP-43’s LCD may disrupt axonal transport. Our data show that mutations which alter TDP-43 net charge exhibit defects in one or more measures of RNP transport. ALS-associated Q343R mutant, for example, shows defects in the ratio of anterograde vs. retrograde motility (Figure 1D), reduced anterograde run lengths and net transport distance (Figure 3). Likewise, N390D mutation reduces the anterograde net velocity of RNP granules (Supplementary Figure S3A). Substitutions of R293A also show profound defects in long-range, directed motility, anterograde and retrograde net velocity, and net transport distance (Figures 5, 6). However, these defects could be due to changes in net charge and/or disruption of the RGG at this site. R293K substitutions also disrupt the RGG but preserve the positive charge at this residue. RNP granules containing R293K also show significantly impaired long-range, directed motility (net distance ≥10 μm; Figure 5A), but display fewer defects compared to R293A (Figures 5D,E). These data suggest both net charge as well as the RGG may regulate RNP granule transport, but additional studies will be required to elucidate the precise role of charge vs. the RGG motif. The reduced motility of RGG mutants raise the possibility that RGG- and/or RNA-dependent protein interactions (i.e., assembly with motors) are disrupted, leading to defects in RNP granule motility. Our data showing that TDP-43 RRM1/RRM2 mutants also display profound reductions in RNP motility (Figure 7) lend support to this possibility.

Regular spacing of pi-pi, hydrophobic and aromatic residues, and arginine-mediated interactions is critical for TDP-43 phase separation and determining phase dynamics (Schmidt et al., 2019). In agreement with this, we observed that aromatic residues in specific regions of the LCD (IDR1, LARKS, or IDR2) are essential for RNP granule transport. Loss of aromatic residues in F313L, F313-6L, F367L, and F397L compromise anterograde (F313L, F397L), retrograde transport (F367L) or both (F313-6L). Schmidt et al., reported that mutating all F to Y does not change the dynamic properties of TDP-43 condensates in HEK-293 cells (Schmidt et al., 2019). An interesting mutant in this context is F313-6Y, which preserves the aromatic residue but reduces the hydrophobicity of this region, yet shows a similar defect in anterograde velocity as F313-6L (Figure 8C). The parsimonious explanation for these observations is that the aromatic ring structure, followed by hydrophobicity, within LARKS are the primary and secondary determinants of TDP-43 RNP granule transport in this region.

In the disordered regions flanking the α-helical domain, we find that F283Y or Y374F substitutions of conserved GFG and SYS motifs, also impair motility, possibly by altering hydrophobicity. Y374F (gain of hydrophobic residue) shows defects in the anterograde and retrograde transport. The possible role of hydrophobic stretches in regulating RNP granule transport is further supported by the F mutants of IDR1 region, though these transport deficits are milder than those seen with Y374T. F283Y and F289Y show defects in net velocity of transport and retrograde transport, respectively (Figure 8).

Further studies will be needed to determine whether differences in the motility of TDP-43 WT and mutant RNP granules can be explained by loss of direct and/or indirect association with the motor proteins kinesin 1 and dynein (Chu et al., 2019). Recently, it was shown that TDP-43 interacts with dynactin and regulates retrograde transport in a cell line (Deshimaru et al., 2021). Therefore, it will be important to understand whether different ALS-linked mutants and TDP-43 substitutions are able to associate with dynactin and other adaptor proteins. Finally, additional studies will be required to clarify whether W334F and Q343R mutations, which fall within the fourth steric zippers (333–343), and Y374F within the fifth steric zipper (370–375) affect TDP-43 aggregation (Guenther et al., 2018).

To summarize, this study is the first to compare the functional impact of ALS-linked TDP-43 mutations on RNP transport in neurons and combine this approach with a systematic mutagenesis study to identify conserved structural elements, aromatic and charged residues that are key determinants of TDP-43 RNP granule transport in the axon. We find that disease-linked mutations and substitutions of aromatics within the α-helical domain and LARKS, structural elements important for phase separation and reversible interactions, respectively, display the most severe defects on TDP-43 RNP granule transport. Specifically, we show that the conserved α-helical domain, phenylalanine residues within LARKS and RGG motif are key determinants of TDP-43 RNP transport, suggesting they may mediate efficient recruitment of motors and adaptor proteins. Further studies will be needed to understand whether aberrant phase transitions of TDP-43 disrupt these interactions, leading to ALS-associated defects in axonal transport and homeostasis.

## Data Availability

The raw data supporting the conclusion of this article will be made available by the authors, without undue reservation.
