# Supplementary material for: Sequence Determinants of TDP-43 Ribonucleoprotein Condensate Formation and Axonal Transport in Neurons
Source: Front Cell Dev Biol. 2022 May 12;10:876893. doi: 10.3389/fcell.2022.876893 (PMC9133736; doi:10.3389/fcell.2022.876893)
Supplement: Supplementary file 1 [file Table1.DOCX]

**Supplementary Table 1: Sequence of the primers used for performing site directed mutagenesis.**

| No. | Primer Name | Primer Sequence |
| --- | --- | --- |
| 1 | αHD (Δ320-340) F | TAATCCAGCCAGCCAGCAGAACCAGTC |
| 2 | αHD (Δ320-340) R | TGCTGGCTGGCTGGATTAATGCTGAAC |
| 3 | Y374F F | AATAACTCTTTTAGTGGCTCTAATT |
| 4 | Y374F R | AGAGCCACTAAAAGAGTTATTTCC |
| 5 | Y374T F | AATAACTCTACTAGTGGCTCTAATT |
| 6 | Y374T R | GAGCCACTAGTAGAGTTATTTCC |
| 7 | W334F F | CTACAGAGCTCTTTCGGTATGATGGGCA |
| 8 | W334F R | CATACCGAAAGAGCTCTGTAGTGCTG |
| 9 | W334L F | CTACAGAGCTCTTTGGGTATGATGGGCA |
| 10 | W334L R | CATACCCAAAGAGCTCTGTAGTGCTG |
| 11 | W385F F | GCAATTGGTTTCGGATCCGCATCCAAT |
| 12 | W385F R | GGATGCGGATCCGAAACCAATTGCTG |
| 13 | W385L F | GCAGCAATAGGCCTTGGATCAGCAT |
| 14 | W385L R | GCTGATCCAAGGCCTATTGCTGCAC |
| 15 | W412F F | AAGTCTTCCGGATTCGGAATGTAGCTCG |
| 16 | W412F R | ACATTCCGAATCCGGAAGACTTAGAATC |
| 17 | W412L F | AAGTCTTCAGGCCTTGGAATGTAGC |
| 18 | W412L R | ACATTCCAAGGCCTGAAGACTTAG |
| 19 | F276Y F | TTAGAAAGATCCGGAAGATATGGTGGTAATC |
| 20 | F276Y R | ACCACCATATCTTCCGGATCTTTCTAACTG |
| 21 | F276L F | AAGTGGACGTTTAGGTGGTAATCCA |
| 22 | F276L R | ATTACCACCTAAACGTCCACTTCTTTC |
| 23 | F283Y F | GTAATCCCGGGGGCTATGGGAATCAG |
| 24 | F283Y R | ATTCCCATAGCCCCCGGGATTACCAC |
| 25 | F283L F | CAGGTGGCCTAGGGAATCAGGG |
| 26 | F283L R | TGATTCCCTAGGCCACCTGGAT |
| 27 | F289Y F | CAGGGTGGCTACGGTAATAGCAG |
| 28 | F289Y R | CTGCTATTACCGTAGCCACCCTGATT |
| 29 | F289L F | AATCAGGGTGGCCTAGGTAATAGCAGAG |
| 30 | F289L R | CTGCTATTACCTAGGCCACCCTGATTC |
| 31 | F313Y F | GGGATGAACTATGGCGCCTTCAGCATTA |
| 32 | F313Y R | ATGCTGAAGGCGCCATAGTTCATCCCA |
| 33 | F313L F | GGGATGAACCTAGGTGCGTTCAG |
| 34 | F313L R | ACGCACCTAGGTTCATCCCACC |
| 35 | F313-6Y F | GGGATGAACTATGGCGCCTACAGCATTAAT |
| 36 | F313-6Y R | TAATGCTGTAGGCGCCATAGTTCATCCCA |
| 37 | F313-6L F | GGGATGAACCTAGGTGCGCTCAGCATT |
| 38 | F313-6L R | GCTGAGCGCACCTAGGTTCATCCCAC |
| 39 | F367Y F | CCAAACCAAGCTTACGGTTCTGGAAAT |
| 40 | F367Y R | TCCAGAACCGTAAGCTTGGTTTGGCTC |
| 41 | F367L F | CCAAACCAAGCTTTAGGTTCTGGAAAT |
| 42 | F367L R | TCCAGAACCTAAAGCTTGGTTTGGCTC |
| 51 | R293A F | TTTGGTAATTCTGCAGGGGGTGGAGCTGGTTTGGGA |
| 52 | R293A R | TCCACCCCCTGCAGATTACCAAATCCACCCTGATTCCC |
| 53 | R293K F | TTTGGTAATTCGAAAGGGGGTGGAGCTGGTTTGGGA |
| 54 | R293K R | TCCACCCCCTTTCGAATTACCAAATCCACCCTGATTCC |
| 55 | A90V F | GATGAGACAGATGTCTCGAGTGCAGTGAAAGTG |
| 56 | A90V R | CACTTTCACTGCACTCGAGACATCTGTCTCATC |
| 57 | G294V F | GGATTTGGTAATTCTAGAGTGGGTGGAGCT |
| 58 | G294V R | AGCTCCACCCACTCTAGAATTACCAAATCC |
| 59 | A315T F | ATGAACTTTGGTACCTTCAGCATTAAT |
| 60 | A315T R | ATTAATGCTGAAGGTACCAAAGTTCAT |
| 61 | Q343R F | ATGTTAGCCTCTAGACAGAACCAGTCA |
| 62 | Q343R R | TGACTGGTTCTGTCTAGAGGCTAACATGCC |
| 63 | A382T F | AATTCTGGTGCAACGATCGGTTGGGGATCA |
| 64 | A382T R | TGATCCCCAACCGATCGTTGCACCAGAATT |
| 65 | N390D F | GGATCAGCTAGCGATGCAGGGTCGGG |
| 66 | N390D R | ACCCTGCATCGCTAGCTGATCCCCA |
| 67 | S393L F | TCCAATGCAGGCCTGGGCAGTGGTT |
| 68 | S393L R | AACCACTGCCCAGGCCTGCATTGGAT |
| 69 | αHD (Δ320-330) F | AGCATTAATCCACAGAGCAGTTGGGGTATG |
| 70 | αHD (Δ320-330) R | CAACTGCTCTGTGGATTAATGCTGAACGCA |
| 71 | F147-9L F | CATTCAAAGGGGCTAGGCCTAGTTCGTTTT |
| 72 | F147-9 R | AAAACGAACTAGGCGTAGCCCCTTTGAATG |
| 73 | F229-31L F | CCATTCAGGGCGCTAGCACTAGTTACATTT |
| 74 | F229-31LR | AAATGTAACTAGTGCTAGCGCCCTGAATGG |
