# Supplementary material for: Sequence Determinants of TDP-43 Ribonucleoprotein Condensate Formation and Axonal Transport in Neurons
Source: Front Cell Dev Biol. 2022 May 12;10:876893. doi: 10.3389/fcell.2022.876893 (PMC9133736; doi:10.3389/fcell.2022.876893)
Supplement: Supplementary file 2 [file Table2.DOCX]

**Supplementary Table 2:** Uniprot accession numbers of TDP-43 sequences from 47 eukaryotic species, used for conservation score analysis.

| **Table S2** | | | |
| --- | --- | --- | --- |
| Uniprot accession number | Species | Uniprot accession number | Species |
| **Q13148** | *Homo sapiens* | **K7FJ67** | *Pelodiscus sinensis* |
| **Q5R5W2** | *Pongo abelii* | **A0A0A0ASR7** | *Charadrius vociferus* |
| **Q921F2** | *Mus musculus* | **G1MZJ1** | *Meleagris gallopavo* |
| **Q5ZLN5** | *Gallus gallus* | **A0A0Q3LZG3** | *Amazona aestiva* |
| **Q28F51** | *Xenopus tropicalis* | **A0A091JMM7** | *Egretta garzetta* |
| **E2QS43** | *Canis lupus familiaris* | **A0A091DKG3** | *Fukomys damarensis* |
| **A0A151N3U6** | *Alligator mississippiensis* | **F7EDX1** | *Ornithorhynchus anatinus* |
| **L9L1M4** | *Tupaia chinensis* | **A0A1U7SU45** | *Tarsius syrichta* |
| **A0A3Q3F2L7** | *Kryptolebias marmoratus* | **A0A2Y9Q513** | *Delphinapterus leucas* |
| **A0A3B3WYB8** | *Poecilia mexicana* | **A0A2K5IA07** | *Colobus angolensis palliatus* |
| **A0A2D0RX33** | *Ictalurus punctatus* | **A0A2K6BKI8** | *Macaca nemestrina* |
| **A0A2R8Q8T5** | *Danio rerio* | **A0A384CER9** | *Ursus maritimus* |
| **U3JXL3** | *Ficedula albicollis* | **A0A2K5WEW9** | *Macaca fascicularis* |
| **A0A091F3Q1** | *Corvus brachyrhynchos* | **I3LNA4** | *Sus scrofa* |
| **A0A1U7R2H0** | *Alligator sinensis* | **A0A2K6A5S4** | *Mandrillus leucophaeus* |
| **A0A087QL47** | *Aptenodytes forsteri* | **A0A452G5Y7** | *Capra hircus* |
| **H0YYI1** | *Taeniopygia guttata* | **G3TD75** | *Loxodonta africana* |
| **A0A452HBC5** | *Gopherus agassizii* | **A0A2Y9RU47** | *Trichechus manatus latirostris* |
| **A0A099Z7N2** | *Tinamus guttatus* | **A0A340XVP8** | *Lipotes vexillifer* |
| **A0A091IJI6** | *Calypte anna* | **A0A2R9C1F8** | *Pan paniscus* |
| **A0A091GT35** | *Cuculus canorus* | **A0A2K5EQY0** | *Aotus nancymaae* |
| **A0A091VW23** | *Nipponia nippon* | **A0A096NBQ0** | *Apio anubis* |
| **A0A218UPU5** | *Lonchura striata domestica* |  |  |
| **U3IED4** | *Anas platyrhynchos platyrhynchos* |  |  |
| **A0A091VBI4** | *Opisthocomus hoazin* |  |  |
