# Supplementary material for: Sequence Determinants of TDP-43 Ribonucleoprotein Condensate Formation and Axonal Transport in Neurons
Source: Front Cell Dev Biol. 2022 May 12;10:876893. doi: 10.3389/fcell.2022.876893 (PMC9133736; doi:10.3389/fcell.2022.876893)
Supplement: Supplementary file 3 [file Table3.DOCX]

**Supplementary Table 3: Summary of TDP-43 variants. and ALS-linked TDP-43 LCD mutations**

| **Mutation** | **fALS/**  **sALS** | **Mutation** | **Cytoplasmic localization** (Buratti, 2015) | **Aggregation** (Buratti, 2015) | **Predicted Phenotype** (Wang et al., 2018) |
| --- | --- | --- | --- | --- | --- |
| **A90V** | **Healthy controls** (Sreedharan et al., 2008; Winton et al., 2008)  **ALS** (Winton et al., 2008)  **Alzheimer’s**  **Disease** (Brouwers et al., 2010) | Non-polar to non-polar | Increased |  | Mutation in NLS may not affect the LLPS properties |
| **G294V** | **sALS** (Del Bo et al., 2009) | Non-polar to non-polar | Increased | Yes. | G promotes fluidity of granules *in vitro.* Loss of glycine could affect fluidity and hence motility. |
| **A315T** | **fALS** (Gitcho et al., 2008; Kabashi et al., 2008) | Non-polar to polar | Increased | Yes.  Increased half life | This residue is close to α helical domain and may affect LLPS. Increased aggregation may hinder motility. |
| **Q343R** | **fALS** (Yokoseki et al., 2008) | Polar uncharged to charged residue |  | Yes. | Loss of Q is associated with reduced hardening of phase separated granules. Change in charge may affect LLPS |
| **A382T** | **fALS** (Kabashi et al., 2008; Del Bo et al., 2009) | Non-polar to polar | Increased |  | Loss of hydrophobicity may affect phase separation |
| **N390D** | **sALS** (Kabashi et al., 2008) | Basic to acidic |  | Yes.  Increased half life | Change in charge may affect LLPS |
| **S393L** | **sALS** (Corrado et al., 2009) | Polar to non-polar |  |  | Loss of serine is associated with reduced hardening of phase separated granules. |
