# Supplementary material for: Sequence Determinants of TDP-43 Ribonucleoprotein Condensate Formation and Axonal Transport in Neurons
Source: Front Cell Dev Biol. 2022 May 12;10:876893. doi: 10.3389/fcell.2022.876893 (PMC9133736; doi:10.3389/fcell.2022.876893)
Supplement: Supplementary file 4 [file Image1.pdf]

Sequence determinants of TDP-43 ribonucleoprotein condensate formation and axonal transport in neurons.

Sonali S. Vishal, Denethi Wijegunawardana, Muthu Raj Salaikumaran, Pallavi P. Gopal

Supplementary Information

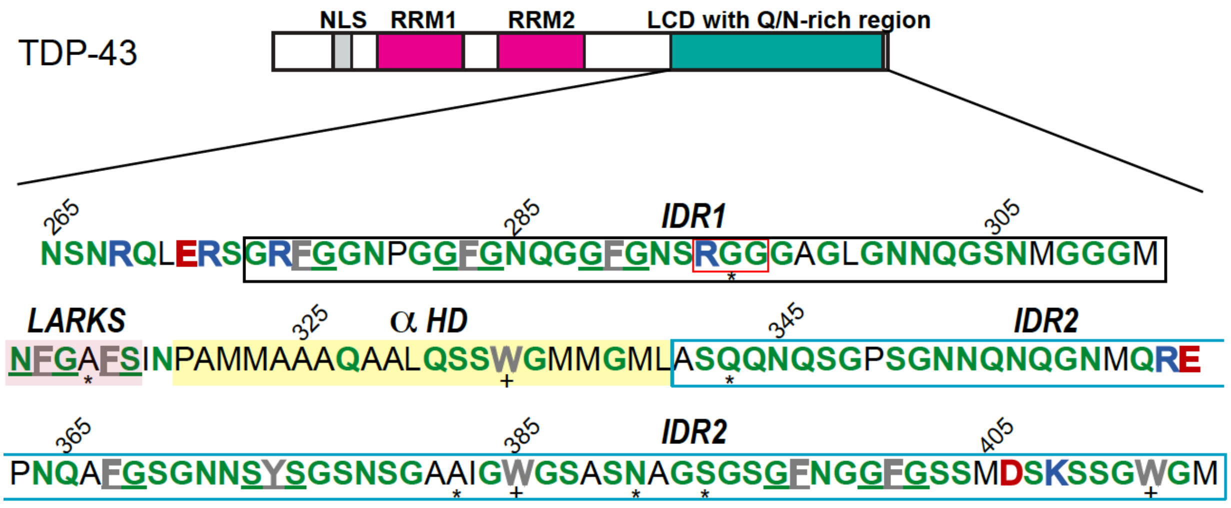

Supplementary Figure 1. Schematic representation of TDP-43 protein highlighting different domains and the amino acid sequence of the LCD. The amino acid residues are color coded based on their structure, charge and polarity, *viz.*, green indicates polar residues, gray indicates aromatic residues, red indicates negatively charged residues and blue indicates positively charged residues. The yellow highlighted region in the amino acid sequence constitutes the conserved α-helical region and the pink highlighted region is LARKS. IDR1 and IDR2 domain are outlined with black and blue boxes, respectively. The different ALS-linked mutants in the present study are denoted with an asterisk (*). Different motifs containing aromatic residues, tyrosine (Y) or phenylalanine (F), such as SYS, GFG, FS and FG/GF, are marked with a green underline (_) and RGG disrupted in this study is labeled with a red box. Tryptophan residues (W) are marked with a plus (+) sign.

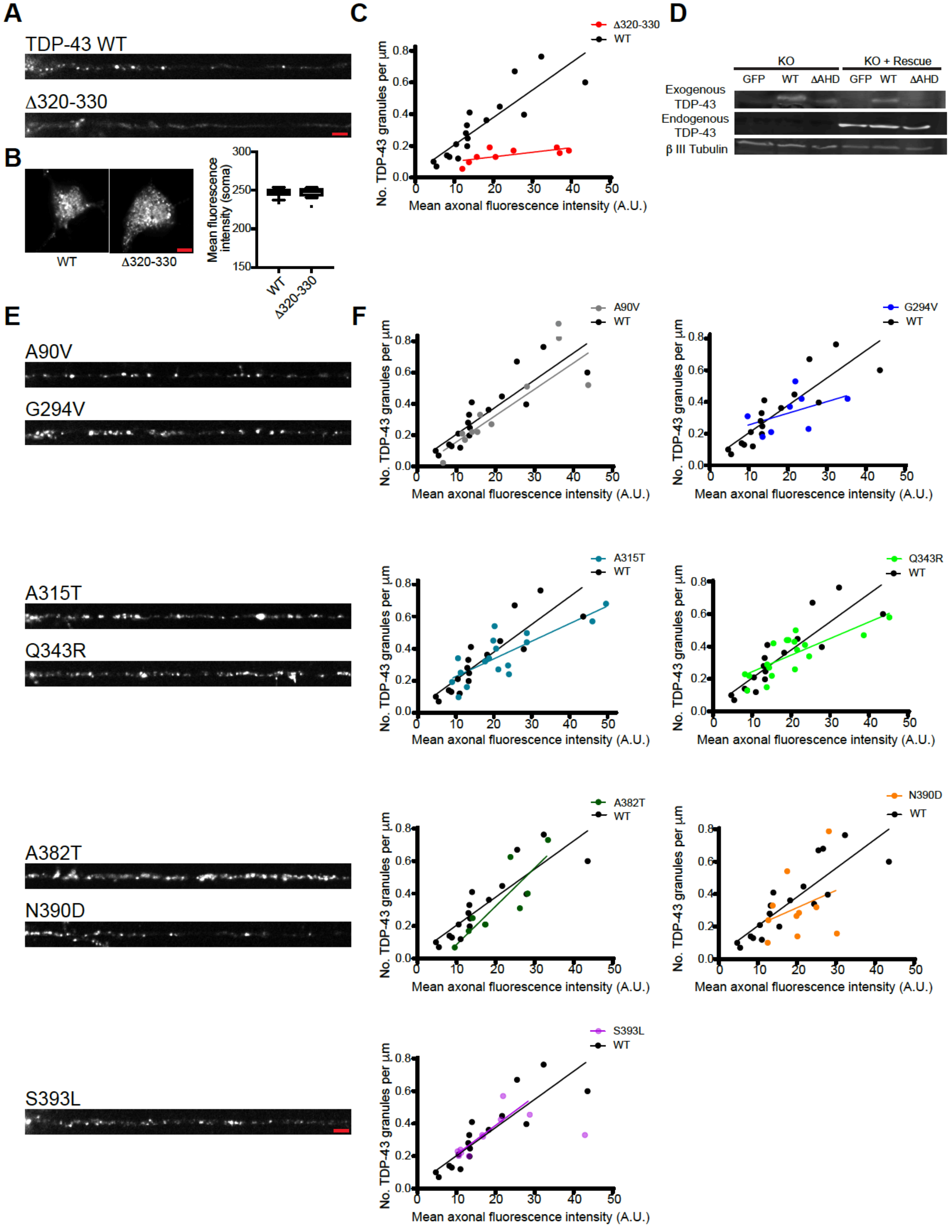

Supplementary Figure 2. TDP-43 Δ320-330 shows reduced RNP condensate formation. (A) TDP-43 RNP granules are observed along the axons of DIV 7-10 primary cortical neurons expressing GFP tagged TDP-43 WT or Δ320-330. Representative images of TDP-43 WT or Δ320-330 RNP granules

are shown. Scale bar, shown in red, corresponds to 3μm. **(B)** Representative images of the soma of primary cortical neurons expressing GFP tagged TDP-43 WT or Δ320-330. Quantification of mean fluorescence intensity of cell soma indicates similar expression level; scale bar = 5μm. Images were acquired at identical laser power, exposure, and sensitivity settings. Total fluorescence intensity of cell body was determined in NIH/Fiji; images were converted to 8 bit and after thresholding average fluorescence was determined. **(C)** The graph shows the number of TDP-43 WT or Δ320-330 RNP granules/μm as a function of GFP fluorescence intensity, which served as a measure of TDP-43 protein expression. A positive correlation is shown for WT TDP-43 (slope = 0.017 ± 0.002; $R^2 = 0.76$), which indicates that the number of RNP granules formed in the axon increases as expression level increases. The slope of this relationship is significantly reduced for TDP-43 Δ320-330 (slope = 0.003 ± 0.001; $R^2 = 0.47$). **(D)** Western blot of TDP-43 knockout (KO) HeLa cell line and KO + rescue line expressing GFP plasmid, GFP TDP-43 WT, or GFP TDP-43 Δ320-330. Lysates were probed with antibodies to TDP-43 and betaIII-tubulin (loading control). **(E)** Representative images of axons from primary cortical neurons expressing different ALS associated TDP-43 mutants; scale bar, shown in red, corresponds to 3μm. **(F)** The number of TDP-43 WT or ALS mutant RNP granules/μm is plotted as a function of TDP-43 WT or mutant GFP fluorescence intensity (estimate of expression level). Simple linear regression analysis was used to determine statistically significant differences of the slope between WT and mutant TDP-43 samples (**A90V** slope = 0.017 ± 0.003, $R^2 = 0.77$; **G294V** slope = 0.007 ± 0.006, $R^2 = 0.23$; **A315T** slope = 0.011 ± 0.002, $R^2 = 0.65$; **Q343R** slope = 0.010 ± 0.002, $R^2 = 0.58$; **A382T** slope = 0.024 ± 0.005, $R^2 = 0.73$; **S393L** slope = 0.018 ± 0.004, $R^2 = 0.76$; **N390D** slope = 0.011 ± 0.011, $R^2 = 0.10$) . For clarity of presentation, TDP-43 WT and each ALS linked mutant are shown on separate graphs. All images were acquired on a spinning disk confocal microscope with constant laser power and exposure settings. Data is from N=3 independent experiments, with n = 10-18 neurons analyzed per condition. # $p < 0.1$, * $p < 0.05$ and *** $p < 0.0005$.

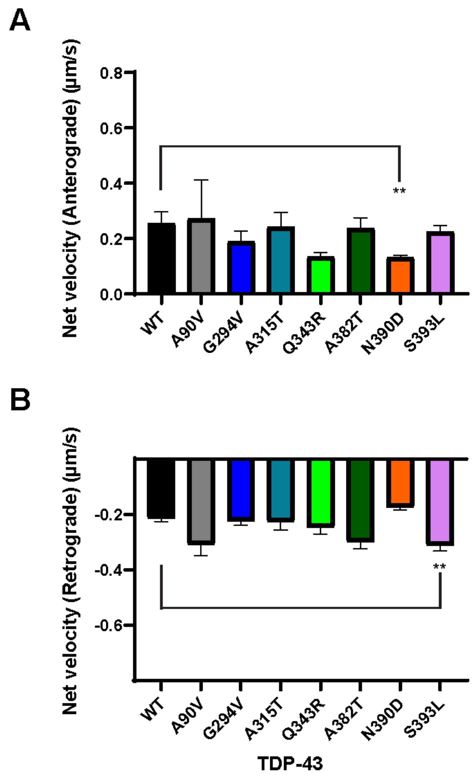

Supplementary Figure 3. TDP-43 N390D shows significantly lower anterograde net velocity. (A) The net velocity (µm/s) for each of the motile WT TDP-43 or ALS mutant TDP-43 granule moving in anterograde direction is determined using custom semi-automated analysis in MATLAB software and the average net velocity (mean ± SEM) is plotted. (WT *n*=75, A90V *n*=16, G294V *n*= 37, A315T *n*=22, Q343R *n*=16, A382T *n*=30, N390D *n*= 87 and S393L *n*=32). **(B)** The net velocity (µm/s) for each of the motile WT TDP-43 or ALS mutant TDP-43 RNP granule moving in retrograde direction is determined using MATLAB software and the average net velocity is plotted (WT *n*=98, A90V *n*=27, G294V *n*=68, A315T *n*=39, Q343R *n*=49, A382T *n*=71, N390D *n*=76 and S393L *n*=78). *n* represents number of motile tracks from 12-16 neurons for TDP-43 mutant or WT TDP-43 from 3 independent experiments. Error bars represent standard error mean. One way ANOVA (Kruskal-Wallis test with Dunn's correction for multiple comparisons) was used to determine the statistically significant difference among different samples. ** $p < 0.01$.

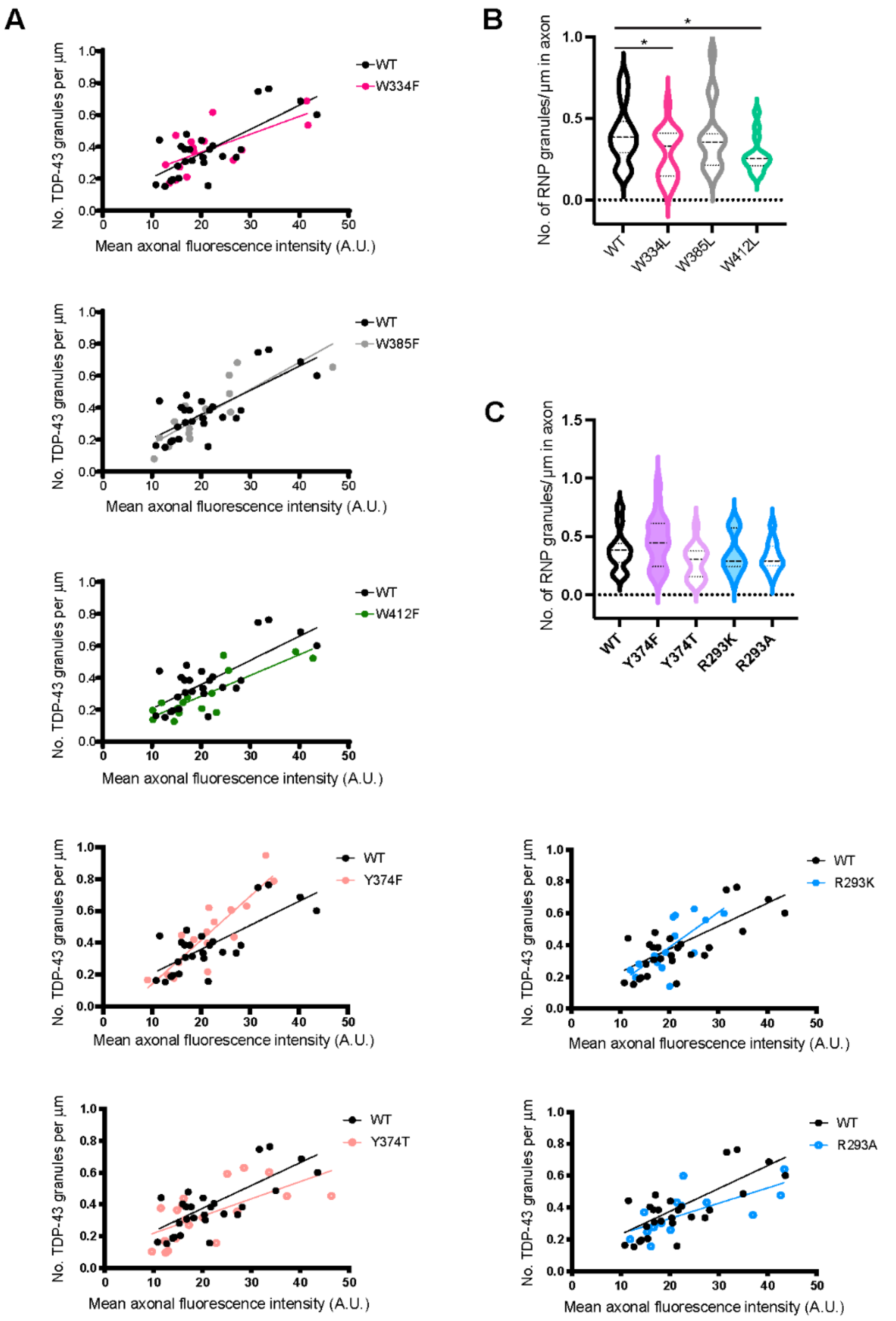

Supplementary Figure 4. (A) The number of TDP-43 WT or W/Y/R mutant RNP granules/µm is plotted as a function of TDP-43 WT or mutant GFP fluorescence. Simple linear regression analysis was used to test for statistically significant differences in the slope between WT and mutant TDP-43 samples (**WT** slope = 0.015 ± 0.003, R^2 = 0.56; **W334F** slope = 0.011 ± 0.003, R^2 = 0.47; **W385F** slope = 0.017 ± 0.003, R^2 = 0.69; **W412F** slope = 0.013 ± 0.002, R^2 = 0.70; **Y374F** slope = 0.028 ± 0.004, R^2 = 0.77; **Y374T** slope = 0.011 ± 0.003, R^2 = 0.40; **R293K** slope = 0.022 ± 0.006, R^2 = 0.52;

R293A slope = 0.010 ± 0.003, $R^2 = 0.49$). For clarity of presentation, TDP-43 WT and each mutant are shown on separate graphs. **(B)** Violin plots showing number of RNP granules per μm length of axon for WT TDP-43 and W TDP-43 mutants or (**C)** Y/R TDP-43 mutants. One way ANOVA (Kruskal-Wallis test with Dunn's correction for multiple comparisons) is used to determine statistically significant differences among different samples. Data is from N=3 experiments from *n*=12-16 neurons per condition. * $p < 0.05$.

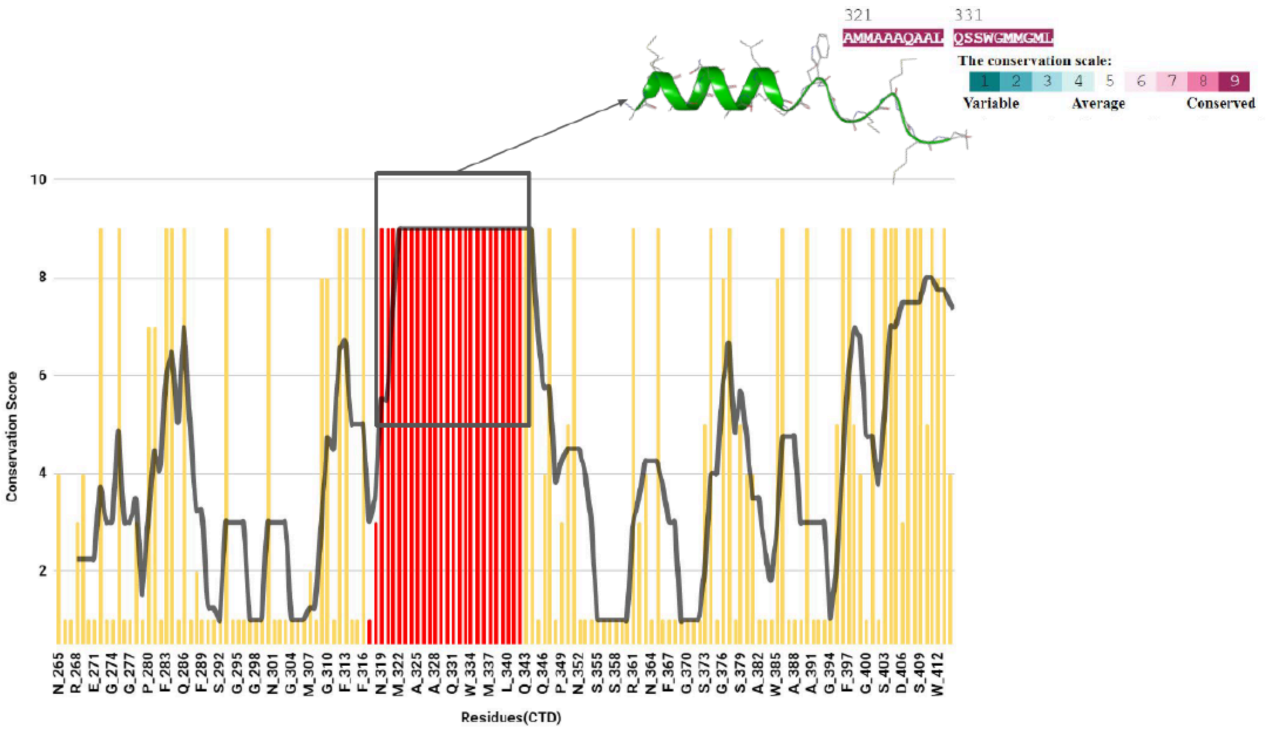

Supplementary Figure 5. The predicted conservation score for TDP-43 amino acids at the C-terminus obtained from 47 eukaryotic species. The x-axis denotes C-terminal residues, while the Y-axis represents the computed conservation score of each residue.

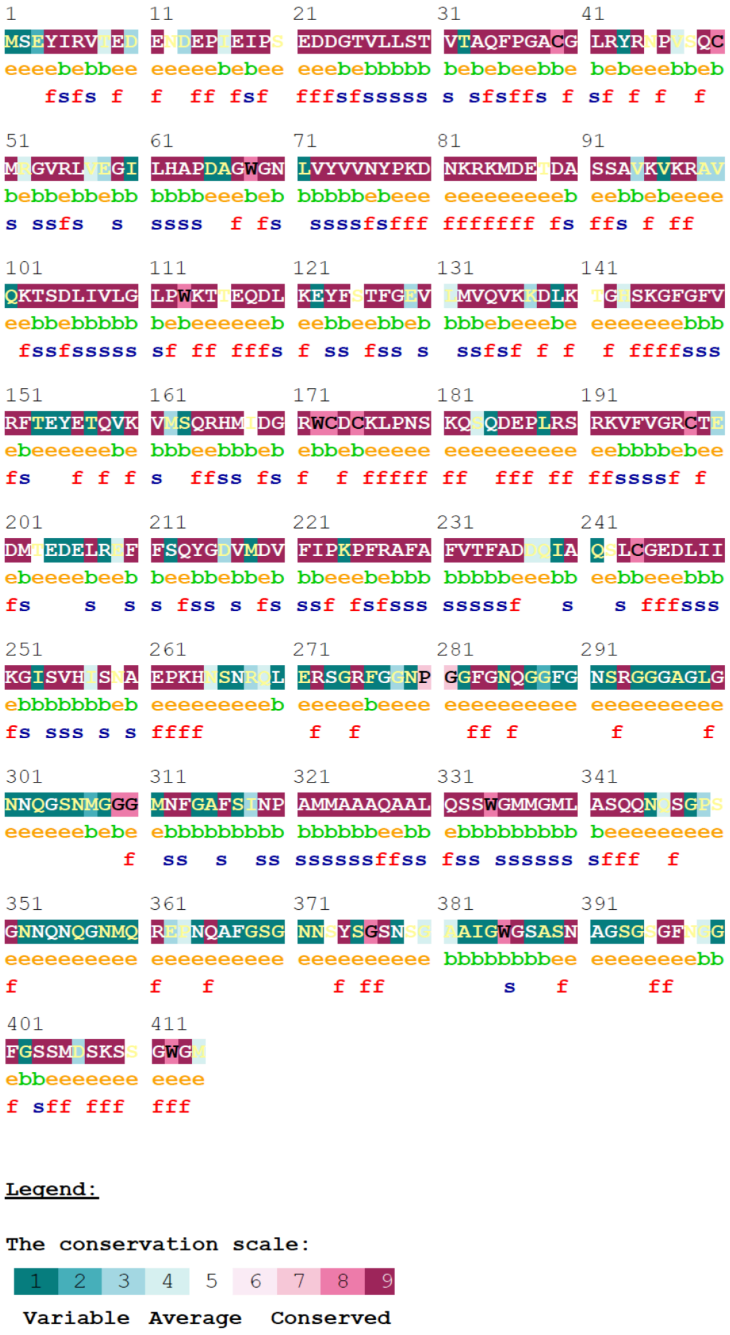

Supplementary Figure 6. TDP-43 conservation scales predicted from 47 eukaryotic TDP-43 sequences. Based on the neural-network algorithm (Berezin *et al.,* 2004), TDP-43 residues predicted to be buried (b), exposed on the surface of the protein (e), structurally (s), or functionally (f) significant are mentioned in the second and third rows.

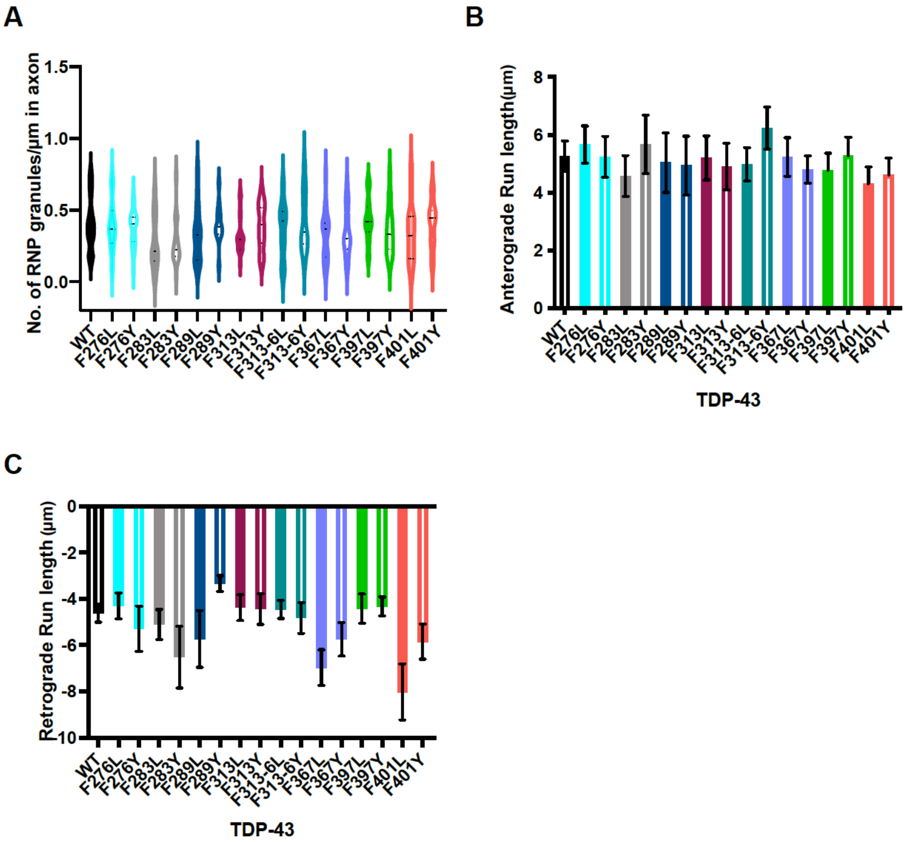

Supplementary Figure 7. Substitutions of phenylalanine residues in LCD region 276-401 do not affect RNP granule formation. (A) Violin plot of number of TDP-43 RNP granules per µm length of axon for WT TDP-43 or F TDP-43 mutants. **(B)** The anterograde run lengths (µm) of motile WT TDP-43 RNP granules or F TDP-43 RNP mutant granules were determined using custom semi-automated analysis in MATLAB and the resulting run lengths (mean ± SEM) are plotted (*N*=3, WT *n*=94, F276L *n*=59, F276Y *n*= 54, F283L *n*=42, F283Y *n*=20, F289L *n*=24, F289Y *n*= 38, F313L *n*=49, F313Y *n*=34, F313-6L *n*=40, F313-6Y *n*= 76, F367L *n*=31, F367Y *n*=48, F397L *n*=47, F397Y *n*=67, F401L *n*=35, F401Y *n*=55). **(C)** Similarly, retrograde run lengths (µm) of motile WT TDP-43 RNP granules or F TDP-43 RNP mutant granules were determined using MATLAB software and the resulting run lengths are plotted (*N*=3, WT *n*=128, F276L *n*=67, F276Y *n*= 60, F283L *n*=58, F283Y *n*=22, F289L *n*=20, F289Y *n*= 49, F313L *n*=52, F313Y *n*=35, F313-6L *n*=62, F313-6Y *n*= 72, F367L *n*=35, F367Y *n*=44, F397L *n*=45, F397Y *n*=72, F401L *n*=33, F401Y *n*=78). *n* represents number of motile tracks from 3 independent experiments. Error bars represent standard error mean. One way ANOVA (Kruskal-Wallis test with Dunn's correction for multiple comparisons) was used to determine statistically significant differences among samples.
